# Increased apolipoprotein-B:A1 ratio predicts cardiometabolic risk in patients with juvenile onset SLE

**DOI:** 10.1016/j.ebiom.2021.103243

**Published:** 2021-02-24

**Authors:** George A. Robinson, Kirsty E. Waddington, Leda Coelewij, Junjie Peng, Meena Naja, Chris Wincup, Anna Radziszewska, Hannah Peckham, David A. Isenberg, Yiannis Ioannou, Coziana Ciurtin, Ines Pineda-Torra, Elizabeth C. Jury

**Affiliations:** a Centre for Rheumatology Research, Department of Medicine, University College London, Rayne Building, London W1CE 6JF, UK; b Centre for Adolescent Rheumatology Versus Arthritis, Department of Medicine, University College London, Rayne Building, London W1CE 6JF, UK; c Centre for Cardiometabolic and Vascular Science, Department of Medicine, University College London, London W1CE 6JF, UK

**Keywords:** Juvenile-onset systemic lupus erythematosus, Cardiovascular disease, Lipids, CD8+ T cells, Interferon

## Abstract

**Background:**

Cardiovascular disease is a leading cause of mortality in patients with juvenile-onset systemic lupus erythematosus (JSLE). Traditional factors for cardiovascular risk (CVR) prediction are less robust in younger patients. More reliable CVR biomarkers are needed for JSLE patient stratification and to identify therapeutic approaches to reduce cardiovascular morbidity and mortality in JSLE.

**Methods:**

Serum metabolomic analysis (including >200 lipoprotein measures) was performed on a discovery (n=31, median age 19) and validation (n=31, median age 19) cohort of JSLE patients. Data was analysed using cluster, receiver operating characteristic analysis and logistic regression. RNA-sequencing assessed gene expression in matched patient samples.

**Findings:**

Hierarchical clustering of lipoprotein measures identified and validated two unique JSLE groups. Group-1 had an atherogenic and Group-2 had an atheroprotective lipoprotien profile. Apolipoprotein(Apo)B:ApoA1 distinguished the two groups with high specificity (96.2%) and sensitivity (96.7%). JSLE patients with high ApoB:ApoA1 ratio had increased CD8+ T-cell frequencies and a CD8+ T-cell transcriptomic profile enriched in genes associated with atherogenic processes including interferon signaling. These metabolic and immune signatures overlapped statistically significantly with lipid biomarkers associated with sub-clinical atherosclerosis in adult SLE patients and with genes overexpressed in T-cells from human atherosclerotic plaque respectively. Finally, baseline ApoB:ApoA1 ratio correlated positively with SLE disease activity index (r=0.43, p=0.0009) and negatively with Lupus Low Disease Activity State (r=-0.43, p=0.0009) over 5-year follow-up.

**Interpretation:**

Multi-omic analysis identified high ApoB:ApoA1 as a potential biomarker of increased cardiometabolic risk and worse clinical outcomes in JSLE. ApoB:ApoA1 could help identify patients that require increased disease monitoring, lipid modification or lifestyle changes.

**Funding:**

Lupus UK, The Rosetrees Trust, British Heart Foundation, UCL & Birkbeck MRC Doctoral Training Programme and Versus Arthritis.

**Research in context**

**Evidence before this study**

Patients with juvenile onset systemic lupus erythematosus (JSLE) have an accelerated risk of developing atherosclerosis, and cardiovascular disease (CVD) is a leading cause of mortality for patients. Despite this, no guidelines exist for the monitoring or management of CVD in SLE patients. The increased risk in JSLE is not explained by traditional CVD risk factors alone but is likely driven by interplay between disease associated chronic inflammation, traditional CVD risk factors (including dyslipidaemia) and risk factors associated with steroid treatment. The continuing high CVD mortality suggests the need for additional therapies targeting lipid metabolism, besides hydroxychloroquine treatment, which has a favourable effect on lipid profile and is widely used in lupus. Cholesterol-lowering drugs (such as statins, which target cholesterol synthesis) have been trialled in SLE patients with mixed outcomes. However, in two major randomised control trials, the Lupus Atherosclerosis Prevention Study (LAPS) in adults and the Atherosclerosis Prevention in Paediatric Lupus Erythematosus (APPLE) trial in children, statins failed to influence atherosclerosis progression. We hypothesised that patient heterogeneity could contribute to the negative results and that the success of future trials could depend on the correct stratification of patients before inclusion into such studies.

We searched PubMed, Web of Science, and Google Scholar for research articles published between Jan 1, 1990, and November 1, 2020, using search terms including “(juvenile-onset) systemic lupus erythematosus”, “atherosclerosis”, “cardiovascular disease”, and “lipoproteins”. We also searched for research articles published in the same time window in rheumatology-specific journals. Published abstracts were excluded from the searches. The earliest referenced article was published in 1993; however, due to modern advancements in cardiovascular research technologies, the majority of articles referenced were more recent (median article year 2015).

**Added value of this study**

This study integrates metabolomic, transcriptomic, immune profile and clinical data from a cohort of patients with the rare disease JSLE and identifies a group of patients with potential increased CVD risk. To our knowledge this is the first study to perform an in depth lipoprotein-based metabolomics study in patients with JSLE. Using unbiased analysis techniques to assess over 220 metabolic biomarkers, including atherogenic and atheroprotective serum lipoproteins, patients with JSLE were stratified into validated groups each with a potential distinct CVD risk and clinical outcome; this was confirmed by cross-validation with markers associated with sub-clinical atherosclerotic plaque in adult SLE patients. Strikingly, the Apolipoprotein-B:Apolipoprotein-A1 (ApoB:ApoA1) ratio was able to predict the high CVD risk group with outstanding accuracy. Furthermore, JSLE patients with High ApoB:ApoA1 ratio had poorer clinical outcomes over 5 year follow-up and a distinct immune profile, characterised by a disrupted CD8+ T cell phenotype which overlapped with CD8+ T-cell profiles identified in patients with atherosclerotic plaques.

**Implications of all the available evidence**

The use of ApoB:ApoA1 in a routine clinical setting could accurately identify young JSLE patients at high cardiometabolic and CVD risk to allow early CVD monitoring for lipid modification and/or dietary intervention where necessary. This could reduce patient mortality outcomes and improve quality of life. The identification of differentially expressed transcriptomic pathways in CD8+ T-cells in high CVD risk patients provides insight into possible therapeutic targets to control inflammation-associated atherosclerotic progression in autoimmunity.

Alt-text: Unlabelled box

## 1 Introduction

Systemic lupus erythematosus (SLE) is a complex autoimmune disorder characterised by loss of immune cell regulation, chronic inflammation and multiple organ damage. Juvenile-onset disease (JSLE) (onset before 18 years) is a rare condition that occurs in up to 20% of patients and is more severe compared to adult-onset SLE [1,2]. Mortality from SLE has improved dramatically over the last 50 years due mainly to improved treatment [3]. However, while deaths attributable to active lupus are reduced, deaths associated with comorbidities including cardiovascular disease (CVD) are still high [4,5]. CVD risk and mortality are particularly increased in JSLE but this risk can only be partially explained by an increased prevalence of traditional risk factors, suggesting a role for inflammation [6]. It is proposed that all patients with SLE, irrespective of age at disease onset, should receive aggressive monitoring and treatment of modifiable CVD risk factors [7], however, no specific guidelines for CVD risk monitoring or management in SLE/JSLE exist. Thus there is an urgent need to find better ways to stratify patients based on their CVD risk and identify adequate therapeutic approaches to decrease the overall cardiovascular morbidity and mortality associated with SLE.

Dyslipidaemia, a conventional risk factor for CVD, is a common feature of patients with both adult and JSLE, and includes elevated triglycerides (TG) and low density lipoprotein (LDL), and reduced high-density lipoprotein (HDL) and apolipoprotein(Apo)-A1 [8], [9], [10], [11], [12]. However, beyond these findings, very little information is available to assess whether defects in lipid metabolism are present in JSLE. Interplay between traditional CVD risk factors (including dyslipidaemia) and risk factors associated with active disease and steroid treatment could contribute to the early, accelerated development of atherosclerosis (chronic inflammation of the large arteries and a major cause of CVD) in JSLE patients [13,14]. However, despite the favorable effect of hydroxychloroquine treatment on lipid profile and its wide use in lupus [15], the continuing high CVD mortality suggests the need for additional therapies targeting lipid metabolism. Cholesterol-lowering drugs (such as statins, which target cholesterol synthesis) have been trialled in SLE patients with mixed outcomes. Some studies show beneficial effects including improved lipid and inflammatory cytokine levels, reduced vascular inflammation, mortality and morbidity in SLE patients [16], [17], [18]. However, in two major randomised control trials, the Lupus Atherosclerosis Prevention Study (LAPS) [19] in adults and the Atherosclerosis Prevention in Paediatric Lupus Erythematosus (APPLE) trial in children [20], statins failed to influence atherosclerosis progression. We hypothesised that patient heterogeneity could contribute to the negative results and that the success of future trials could depend on the correct stratification of patients before inclusion into such studies [21,22] as well as identification of other potential therapeutic targets.

Here, in depth lipoprotein-based metabolomics profiling defined and validated two distinct JSLE patient groups based on their ApoB:ApoA1 ratio, a biomarker previously associated with increased CVD risk [23,24]. Patients with high ApoB:ApoA1 ratios, had a unique immune and transcriptomic signature represented by elevated CD8+ T-cell frequencies, altered expression of genes associated with atherogenic processes including apoptosis, T-cell activation and interferon (IFN) signalling and a more active disease trajectory over time. Collectively, these findings could help identify JSLE patients at greatest CVD risk and select those patients who would benefit from targeted lipid modifying therapies.

## 2 Materials and methods

### 2.1 Study design and patients

#### 2.1.1 Patients

Peripheral blood was collected from JSLE patients fulfilling The American College of Rheumatology (ACR) classification criteria for lupus (1997) [25] or the Systemic Lupus International Collaborating Clinics (SLICC) criteria (2012) [26], attending a young adult or adolescent rheumatology clinic at University College London Hospital (UCLH) between Sept 5, 2012, and March 7, 2018. Additional inclusion criteria included JSLE samples with more than 10 million peripheral blood mononuclear cells (PBMCs)/sample. Full details of inclusion and exclusion criteria and relevant protocol details are in Supplementary Materials Page 2. Two cohorts were collected: a discovery cohort (n=31) and a validation cohort (n=31). Detailed patient and disease characteristics (including demographics, age at onset, disease duration, clinical and serological parameters, and medication) were collected from medical records and through questionnaires at the time of blood sampling (Table 1). Disease activity was calculated using the SLE Disease Activity Index-2000 (SLEDAI-2000). A score greater than 4 was used to stratify more active patients as our cohort comprised JSLE patients with milder disease activity at the time of inclusion in the study [27]. Lupus Low Disease Activity State (LLDAS) was recorded for all patients [28]. Longitudinal disease activity scores were collected for all JSLE patients from baseline (time of metabolomics and immune-phenotype analysis) to most recent clinical appointment, comprising 3-7 years follow-up (mean duration=4.9 years, mean number of visits/patient=17.1). These data were analysed by group averages over time and/or by spaghetti plots for individual patient trajectory. Baseline metabolomics data was correlated with the averages of longitudinal disease activity scores by linear regression. Written, informed consent was acquired from all patients. All information was stored as anonymised data. Patients treated with rituximab and cyclophosphamide within the past year were excluded from the study due to previously reported substantial improvements in CVR measures following effective treatment [29].

**Table 1:** Demographic and clinical table of discovery and validation JSLE patient cohorts.

Table 1	JSLE (discovery)	JSLE (validation)
Total number	31	31
Female:Male	21:10	30:1
Age, mean (range, SD)	19 (14-25, 2.92)	19 (13-24, 2.61)
BMI, median (IQR)	21.51 (20.27- 26.46)	23.06 (20.44- 25.88)
Ethnicity, number (%):		
White	11 (35%)	8 (26%)
Asian	11 (36%)	9 (29%)
Black	7 (23%)	11 (35%)
Other/unknown	2 (6%)	3 (10%)
Disease characteristics		
Age of diagnosis, mean (range, SD)	11.7 (0-18, 4.15)	13 (8-18, 3.30)
Disease duration, mean (range, SD) (years)	7.5 (0-21, 4.68)	6.4 (0-14, 3.81)
SLEDAI-2000, median (IQR)	2 (0-4))	0 (0-2)
SLEDAI-2000 >4, number (%)	7 (23%)	1 (3%)
Current organ involvement, n (%):		
Neurological	2 (6.45%)	9 (29%)
Serositis	7 (23%)	0 (0%)
Cutaneous	30 (97%)	25 (81%)
Haematological	13 (42%)	12 (39%)
Musculoskeletal	26 (84%)	24 (77%)
Renal	9 (29%)	9 (29%)
Serology [median (IQR)]:		
dsDNA (IU/mL) (NR=<50)	53.5 (3-332)	21 (6-60)
Positive ENA (number, %)	18 (58%)	18 (58%)
Anti-CL IgM (MPL) (NR=0-10)	2.7 (1.1-4.2)	1.75 (1.1-3.93)
Anti_CL IgG (GPL) (NR=0-20)	1.7 (0.6-4)	1.2 (0.35-1.9)
hsCRP (mg/L) (NR<5mg/L)	1 (0.6-2.58)	0.7 (0.6-1.63)
C3 (g/L) (NR=0.9-1.8) (mean (range, SD))	0.97 (0.33-1.64, 0.33)	0.94 (0.57-1.47, 0.23)
LC (10^9^/L) (NR=1.3-3.5)	1.47 (1.2-2.09)	1.08 (0.91-1.25)
ESR (mm/hr) (NR=<20)	10 (2-127)	11 (2-94)
Urine protein:creatinine ratio (mg/mmol) (NR=0-13)	8 (6-13)	8 (5.75-10)
Clinical lipids [median (IQR)]:		
Cholesterol (NR<5mmol/L)	3.95 (3.48-4.2)	4 (3.4-4.9)
Triglycerides (NR<3mmol/L)	0.8 (0.58-1.23)	0.8 (0.5-1)
HDL-C (NR>1mmol/L)	1.45 (1.25-1.7)	1.4 (1.1-1.7)
LDL-C (NR<3mmol/L)	2 (1.6-2.23)	2.2 (1.5-2.6)
Cholesterol:HDL (NR<4)	2.55 (2.2-3.35)	2.9 (2.5-3.4)
Non-HDL-C (NR<4mmol/L) (Nightingale)	2.5 (2.17-3.03)	2.5 (2.01-2.78)
Current treatment [n (%)]:		
Prednisolone	16 (52%)	11 (35%)
Prednisolone dose (mg), mean (SD)	4.129 (5.379)	2.935 (4.566)
Hydroxychloroquine	27 (87%)	29 (94%)
Methotrexate	3 (10%)	4 (13%)
Mycophenolate mofetil	15 (48%)	9 (29%)
Azathioprine	7 (23%)	8 (26%)
Vitamin D	7 (23%)	6 (19%)
Past rituximab treatment (%)		
Rituximab in the last year	0 (0%)	0 (0%)
Rituximab ever	3 (8.6%)	1 (1%)
Cyclophosphamide in the last year	0 (0%)	0 (0%)

For patients the SLE Disease Activity Index (SLEDAI)-2000 was calculated, a score greater than 4 represents active disease [27]. Normal ranges for lipid measures are relevant for healthy adults. Abbreviations: NR: Normal ranges, BMI: Body mass index, SLEDAI: Systemic Lupus Erythematosus Disease Activity Index, ENA: Extractable nuclear antigens, Anti-CL: Anti-cardiolipin, dsDNA: Anti-double-stranded-DNA antibodies, hsCRP: high sensitivity C-reactive protein, C3: Complement component 3, LC: Lymphocyte count, ESR, erythrocyte sedimentation rate, HDL-C: High density lipoprotein cholesterol, LDL-C: Low density lipoprotein cholesterol.

#### 2.1.2 Sample size

This was an exploratory study based on the number of patients available fitting the inclusion/exclusion criteria. No outliers were excluded.

#### 2.1.3 Ethics approval

This study was approved by the London-Harrow research Ethics Committee 11/LO/0330 and the London - City & East Research Ethics Committee 15-LO-2065.

### 2.2 Metabolomics

Serum metabolomics analysis was performed using nuclear magnetic resonance (NMR) spectroscopy by Nightingale Health (https://nightingalehealth.com/, date last accessed September 2020) [30] (Supplementary Table 1 for list of metabolites).

### 2.3 Flow cytometry

#### 2.3.1 Immunophenotyping

2 × 10^6^ PBMCs were stained with fixable blue dead cell stain (ThermoFisher) or Zombie NIR™ Fixable Viability Kit (Biolegend) in PBS, followed by washes and surface marker staining in Brilliant™ Stain Buffer (BD) with antibodies (Biolegend unless stated): CD3-BV786 (317330), CD4-BUV395 (BD) (563550), CD8-BV421 (301036), CD27-AF700 (356416), CD45RA-PE-Cy7 (304126), CD25-PEdazzle594 (356126), CD127-BV711 (351328), iTCR-PE (342904), CD19-AF488 (302219), IgD-BV510 (348220), HLA-DR-BV786 (307642), CD38-BV421 (356618), CD14-BV711 (301838), CD16-PEdazzle594 (302054), CD303-PerCP-Cy5.5 (354210) followed by subsequent washes and fixation in 2% PFA. Stains and washes were carried out in Biolegend cell staining buffer. Data was acquired using a BD LSRFORTESSA X-20 flow cytometer (1-2 × 10^6^ cells per sample) and analysis performed using FlowJo Single Cell Analysis Software (TreeStar). Cytometer Setup and Tracking (CS&T) (BD) beads were used to monitor cytometer performance. Application settings were applied prior to compensation to ensure that all immunophenotyping data was comparable over time. Gating strategies are shown in Supplementary Figure 1.

### 2.4 Cell sorting and RNAseq

#### 2.4.1 Sorting

PBMCs from 12 JSLE patients were washed in MACS buffer (1X PBS (Sigma), 2% FBS (Labtech) and 1mM EDTA (Sigma)) and stained with Zombie NIR™ Fixable Viability Kit (Biolegend) in PBS followed by staining for 30 minutes with anti-human CD3-BV786 (Biolegend), CD4-BUV395 (BD Biosciences) and CD8-BV421 (Biolegend) in MACS buffer. Samples were sorted in MACS buffer using BD FACSAria flow cytometry cell sorter into collection media (1xPBS, 20% FBS).

#### 2.4.2 RNA preparation

Total RNA was isolated from FACS sorted CD4+ and CD8+ T-cells and using the PicoPure RNA isolation kit (Applied Biosystems). RNA integrity was confirmed using Agilent's 2200 Tapestation. UCL Genomics (London, UK) performed cDNA library preparation using the NEB RNA Ultra II Directional assay with Poly(A) mRNA workflow (p/n E7760) according to manufacturer's instructions. Briefly, mRNA was isolated from total RNA using Oligo dT beads to pull down poly-adenylated transcripts. The purified mRNA was fragmented using chemical hydrolysis (heat and divalent metal cation) and primed with random hexamers. Strand-specific first strand cDNA was generated and “A-tailed” at the 3’ end. Full length xGen adaptors (IDT), containing unique 8bp dual sample specific indexes, a unique molecular identifier and a T overhang are ligated to the A-Tailed cDNA. Successfully ligated cDNA molecules were then enriched with limited cycle PCR.

#### 2.4.3 Sequencing

Sequencing and quality control analysis was performed by UCL Genomics. Libraries to be multiplexed in the same run were pooled in equimolar quantities, calculated from Qubit and Bioanalyser fragment analysis. Samples were sequenced on the NextSeq 500 instrument (Illumina), with a 43bp paired end protocol.

#### 2.4.4 RNAseq computational analysis

Reads were demultiplexed and converted to fastq files by UCL Genomics using Illumina's bcl2fastq Conversion Software v2.19. The rest of the analysis pipeline was performed using Partek Flow software v9.0.20.0426 using the RNA-seq toolkit. Sequence reads were aligned using STAR v2.5.3a to the human human Hg38 reference genome. The reads where then filtered to removed singletons and unaligned reads. Gene count abundance was quantified using Partek E/M Annotation Model to human Hg38 - Refseq transcripts 92 2019-11-01. Samples were grouped by ApoB:ApoA1 ratio according to the cut off value obtained in Fig. 3. To establish differences in gene expression between groups, DESeq2-3.5 analysis was performed using Wald hypothesis test and parametric fit type with FDR step-up.

Pathway enrichment, network analysis, comparative heat maps and circos plots were was performed using the web-based portal Metascape [http://metascape.org, date last accessed July 2020] on a list of statistically significant (Fold change 1.3, p<0.01) differentially expressed genes in CD4+ and CD8+ T-cells in high vs low ApoB:ApoA1 ratio JSLE patients. This provided a comprehensive gene list annotation curated via KEGG, Reactome and gene ontology databases. Venn diagrams were generated with BioVenn [http://www.biovenn.nl/index.php, date last accessed July 2020].

### 2.5 Statistical analysis

Data analysis plan is shown in Supplementary Fig. 2. Statistical analysis was performed using GraphPad Prism-7. Data was tested for normal distribution and parametric/non-parametric tests were used accordingly. Unpaired and paired t-tests and One-way ANOVA (Turkey's post-hoc test) were used as appropriate. Linear regression was performed by Pearson correlation using a 95% confidence interval. Multiple testing was accounted for using the Holm-Sidak correction of p-values. Receiver operating characteristic (ROC) curve analysis was performed using GraphPad Prism-7. C-statistic was reported as area under curve (AUC) for all ROC analysis.

#### 2.5.1 Hierarchical clustering

Data analysed by Pearson's correlation using z-score transformed raw data values calculated in Microsoft Excel 2010 using the equation: *((Individual Value-Population Mean)/(Population Standard Deviation–Square Root of the Total Sample Number))*. Clustering heatmaps produced using MultiExperiment Viewer (MeV)*.* ClustVis web-tool [31] was used to perform principle component analysis (PCA).

#### 2.5.2 Logistic regression

performed in RStudio [32] on each individual metabolomic biomarker or immune cell frequency and adjusted for demographic (age, sex, ethnicity, BMI) clinical (disease duration, disease activity (SLEDAI-2000), dsDNA autoantibodies, high sensitivity C-reactive protein (hsCRP), complement-C3 (C3), lymphocyte count) and treatment parameters. Logistic regression of metabolomics data was visualised using the R package foresplotNMR [33].

#### 2.5.3 Role of the funding source

The funders played no part in the collection, analysis, and interpretation of data; in the writing of the report; and in the decision to submit the paper for publication.

## 3 Results

### 3.1 JSLE patients can be stratified based on complex serum lipoprotein profiles

**Fig. 1:**
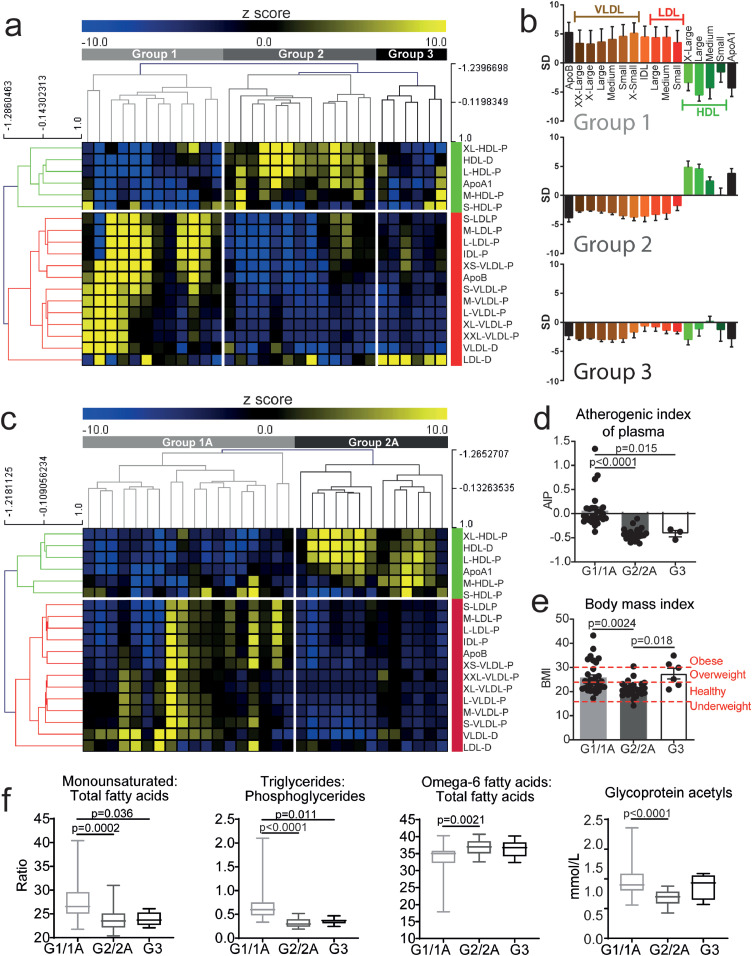
*JSLE patient stratification by lipoprotein profile reveals unique and validated patient groups***.** Metabolomic analysis was performed on serum from a discovery cohort (n=31, **a-b**) and validation cohort (n=31, **c**). **(a)** Heat map showing unbiased hierarchical clustering of lipoprotein particle (P) concentrations and diameters (D) and apolipoproteins (Apo), z-score converted measurements. Groups are labelled; Group-1=light grey, Group-2=dark grey, Group-3=black. Anti- and pro- atherogenic lipoproteins clustered (labelled in green and red respectively). **(b)** Lipoprotein subclasses measurements from Group-1-3 (from A). Standard deviation (SD) units showing deviation from the mean value of the whole study population. Error bars indicate standard error. **(c)** Heat map showing unbiased hierarchical clustering of lipoproteins and apolipoproteins using z-score converted measurements. Two groups identified: Group-1A=light grey and Group-2A=dark grey. Anti- and pro- atherogenic lipoproteins clustered (labelled in green and red respectively). **(d-e)** Discovery and validation JSLE cohort data were combined (Group-1/1A (n=30), Group-2/2A (n=26)) and discovery cohort Group-3 (n=6). **(d)** Atherogenic index of plasma [Log(Triglycerides/HDL-Cholesterol)] and **(e)** Body Mass Index (BMI) of patients. Dashed red lines indicate the cut off BMI values for underweight, healthy, overweight and obese. **(f)** Levels of four metabolites identified previously in SLE patients with pre-clinical plaque [35] in Group-1/1A (n=30), Group-2/2A (n=26)) and discovery cohort Group-3 (n=6). One-way ANOVA. Abbreviations: Apo, apolipoprotein; VLDL, IDL, LDL, HDL, very low, intermediate, low and high-density lipoproteins; XX-large, chylomicrons and extremely large; X-Large, very large; X-small, very small.

Dyslipidaemia was investigated in patients with JSLE using an in-depth lipoprotein-based metabolomics platform (Discovery cohort, Table 1, and Supplementary Table 1 for metabolite list and Supplementary Fig. 2 for analysis plan). Unbiased hierarchical clustering identified three distinct clusters based on lipoprotein particle concentrations and diameters (Fig. 1a). Group-1 was characterised by reduced serum HDL and elevated very low-density lipoprotein (VLDL), intermediate density lipoprotein (IDL) and LDL particles - suggestive of an atherogenic metabolomic profile; Group-2 had elevated HDL and reduced VLDL/IDL/LDL particles – a more atheroprotective profile and; Group-3 had relatively low serum levels of HDL and VLDL/LDL/IDL particles (Fig. 1a and b, Supplementary Table 2).

The robustness of the metabolomic stratification was tested in a second cohort of 31 JSLE patients, recruited with no selection bias other than no rituximab or cyclophosphamide treatment in the previous year (Validation cohort, Table 1). This cohort had a statistically significant increase in female:male ratio and frequency of neurological and serositis manifestations although median SLEDAI-2000 was reduced compared to the discovery cohort. Using the same lipoprotein markers and unbiased hierarchical clustering method, patients clustered into 2 groups (Fig. 1c) (labelled Group-1A and 2A) which reflected the pro- and anti- atherogenic lipoprotein phenotypes observed in Groups-1 and 2 of the discovery cohort (Fig. 1a). Importantly, Group-1 and 1A clustered together, but independently from Group-2 and 2A by PCA (Supplementary Fig. 3). Group-3 was not validated. Despite statistically significant lipid metabolomic differences, there were no statistically significant differences in disease activity score (SLEDAI-2000), age of onset, disease duration or treatment between Group-1 and 2 or Group-1A and 2A (Supplementary Tables 3 and 4). However, although almost all patients in both cohorts had total cholesterol and TG levels within normal clinical ranges, patients in Group-1 and 1A had reduced HDL cholesterol and elevated LDL cholesterol compared to Group-2 and 2A respectively and Group-3 (Supplementary Tables 3 and 4).

For the remainder of this study, patients from Group-1 and 1A (Group1/1A) or Group-2 and 2A (Group-2/2A) were combined between discovery and validation cohorts.

When other measures traditionally associated with CVD risk were examined, patients in combined Groups-1/1A had an increased atherogenic index of plasma (Log(Triglycerides/HDL-Cholesterol) [34]) compared to Groups-2/2A and 3 (Fig. 1d) and an increased BMI compared to Group-2/2A (Fig. 1e and Supplementary Tables 3 and 4), suggesting that JSLE patients in Group-1/1A could have increased CVD risk. In order to verify this, metabolites known to have altered expression in adult SLE patients with established pre-clinical carotid and femoral artery atherosclerotic plaque were examined [35]: namely elevated ratios of monounsaturated:total fatty acids and triglycerides:phosphoglycerides, elevated glycoprotein acetyl levels and reduced ratios of omega-6:total fatty acids. These biomarkers were all statistically significantly altered in Group-1/1A JSLE patients compared to Groups-2/2A and Group-3 (Fig. 1f). Together, these results suggested that metabolomic serum analysis could stratify JSLE patients according to their potential CVD risk.

### 3.2 ApoB:ApoA1 ratio could be an independent biomarker for JSLE patient group stratification

**Fig. 2:**
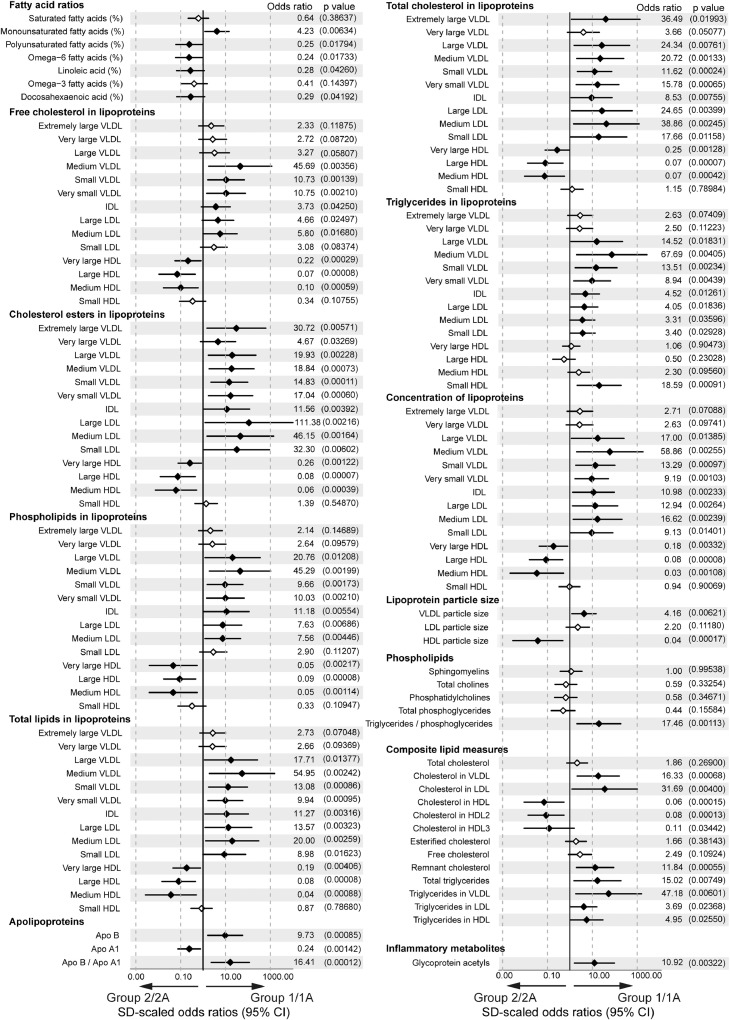
*Lipoprotein stratified groups had distinct lipid and inflammatory metabolite profiles***.** Forest plot showing OR and 95% CI of serum metabolites between Group-1/1A (n=30) and Group-2/2A (n=26) adjusted for age, sex, ethnicity, BMI, disease parameters (CRP, dsDNA, C3, lymphocyte count, disease duration, SLEDAI-2000) and treatment (Hydroxychloroquine, Prednisolone, Mycophenolate Mofetil, Methotrexate, Azathioprine, and Vitamin D). Measurements include specific fatty acids (% of total fatty acids), cholesterol, triglycerides and phospholipids (mmol/l), apolipoproteins (g/l) and in-depth lipoprotein measurements including particle size (nm), concentration and lipid content (mmol/l). Statistically significant differences denoted by solid black diamond; non-statistically significant differences denoted by open diamond. Odds ratio and p values (in brackets) displayed to the right. Associated clinical and demographic parameters affecting metabolite expression are shown in Supplementary Data Table 1. Abbreviations: Apo, apolipoprotein; VLDL, very low-density lipoprotein; IDL, intermediate density lipoprotein; LDL, low density lipoprotein; HDL, high density lipoprotein.

To investigate the metabolomic profiles of each group in more detail, logistic regression analysis was performed comparing combined Groups-1/1A with Groups-2/2A; data was normalised for age, sex, ethnicity, BMI, disease duration, disease activity (SLEDAI-2000), dsDNA autoantibodies, high sensitivity C-reactive protein (hsCRP), complement-C3 (C3), lymphocyte count and treatment (Fig. 2, Supplementary Fig. 4, Data Table 1). A number of differentially expressed lipid metabolites were identified. This included elevated serum concentration of monounsaturated fatty acids and VLDL, IDL and LDL and increased TG, phospholipid and cholesterol ester lipid content in VLDL/IDL/LDL in Group-1/1A patients, whereas elevated HDL and unsaturated fatty acids and altered HDL lipid content (increased phospholipids and cholesterol esters) characterised patients in Group-2/2A (Fig. 2). Interestingly Glycoprotein Acetyl, related with increased cardiovascular mortality risk [36,37], was the only non-lipid metabolite differentially expressed between the groups (Supplementary Fig. 4).

**Fig. 3:**
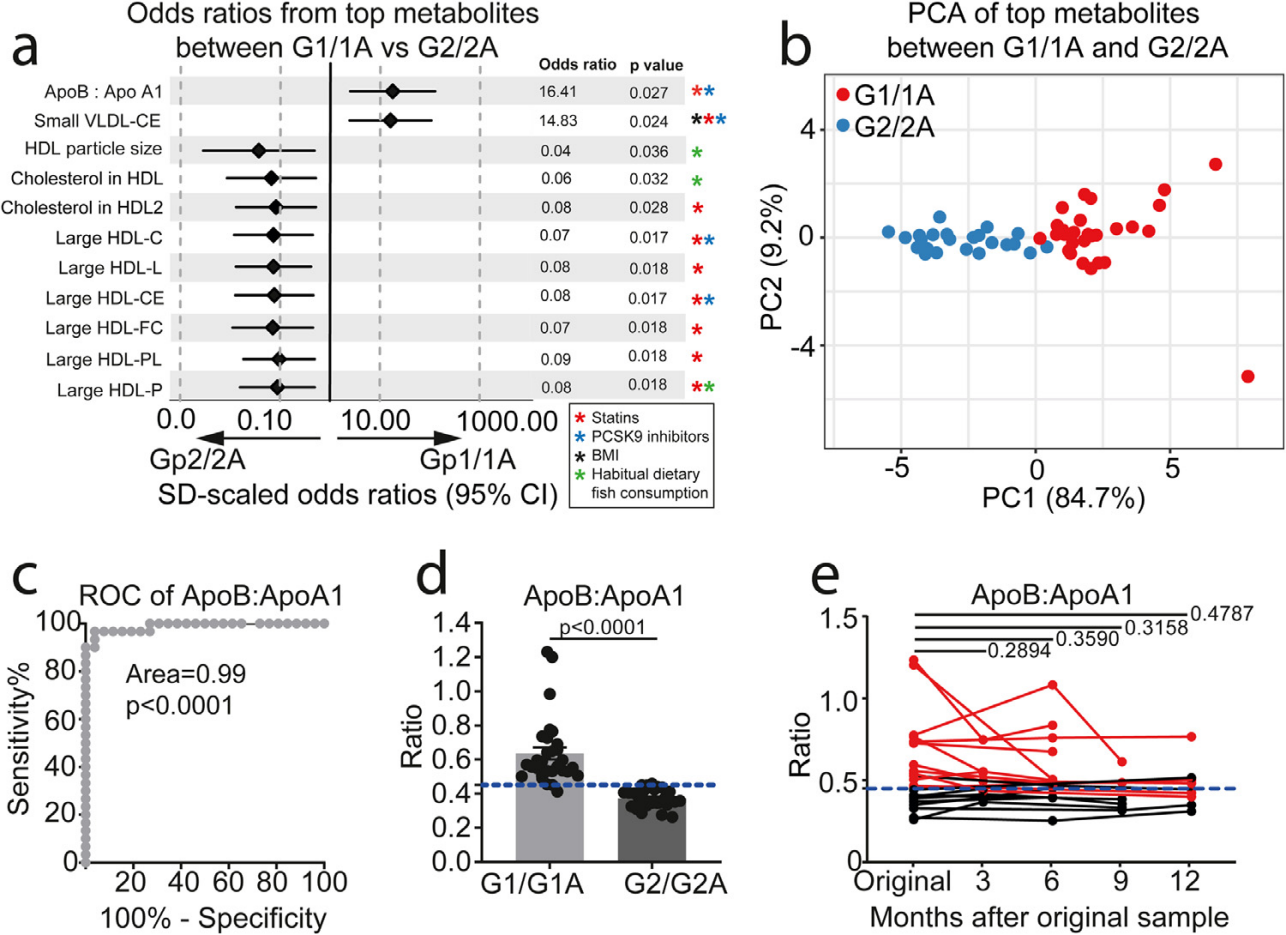
*ApoB:ApoA1 as a clinical biomarker for stratified JSLE groups***. (a)** Forest plot from logistic regression analysis displaying the OR and 95% CI of 11 metabolites that passed p value correction for multiple testing between Group-1/1A (n=30) and Group-2/2A (n=26). Data was normalised for clinical parameters, demographic information, BMI, disease parameters (CRP, dsDNA, C3, lymphocyte count, disease duration, SLEDAI-2000) and treatment (Prednisolone, Hydroxychloroquine, Mycophenolate, Methotrexate, Azathioprine and Vitamin D). Corrected p-values (Holm-Sidak) displayed for each metabolite. Stars represent metabolites that are statistically significantly affected by BMI (Black), statins (Red)(38-40), PCSK9 inhibitors (Blue)(40) and habitual dietary fish consumption (Green)(41). **(b)** Principle component analysis using the 11 statistically significant metabolites between Group-1/1A (n=30, red) and Group-2/2A (n=26, blue) identified in (A). **(c)** ROC curve analysis of the ApoB:ApoA1 ratio of patients in Group-1/1A (n=30) compared to patients in Group-2/2A (n=26). Area under the curve (AUC) displayed. **(d)** ApoB:ApoA1 ratio (higher ratios=higher risk) measured in Group-1/1A (n=30) and Group-2/2A (n=26). Cut-off identified from the ROC analysis displayed as the blue dashed line. Mean, unpaired two-tailed t-tests. **(e)** Longitudinal analysis of ApoB:ApoA1 ratio at 3 month intervals over 12 months (baseline (n=22), 3 months (n=9), 6 months (n=12), 9 months (n=6) and 12 months (n=9)). The cut off identified from the ROC curve analysis is displayed as the blue dashed line. Red points/lines represent patients that started with a baseline ApoB:ApoA1 ratio above the cut off. Paired t-tests.

By applying stringent p value correction for multiple testing to the logistic regression data, 11 metabolites were identified as differentially expressed between Group-1/1A and Group-2/2A in JSLE (Fig. 3a, Supplementary Table 5). This included increased ApoB:ApoA1 ratio and concentration of small (S-)VLDL cholesterol esters (VLDL-CE) associated with Group-1/1A and large (L-)HDL particle concentration and particle size, and L-HDL lipid content (total lipids (L-HDL-L), cholesterol (L-HDL-C), cholesterol esters (L-HDL-CE), Free cholesterol (L-HDL-FC) and phospholipids (L-HDL-PL)) associated with Group-2/2A. In addition, PCA using these combined top 11 differentially expressed metabolites confirmed independent clustering of patients in each group (Fig. 3b) and identified that the ApoB:ApoA1 ratio and L-HDL-L were the top weighted factors driving the cluster separation in PC1 (84.7% of the total variance) (Supplementary Table 6). Of note, the majority of these metabolites can be significantly affected by treatment with statins [38,39] and pro-protein convertase subtilisin/kexin type 9 (PCSK9) inhibitors [40], dietary fish consumption and/or BMI [41] (Fig. 3a-askerisks), suggesting that abnormal serum lipid metabolite profiles in JSLE patients, which are potentially associated with increased CVR, could be modified using available therapies or interventions.

Receiver Operator Characteristic (ROC) analysis of the 11 differentially expressed metabolites confirmed that ApoB:ApoA1 ratio had the highest predictive power to distinguish patients in Group-1/1A from patients in Group-2/2A (AUC=0.99, p<0.0001, specificity=96.2% and sensitivity=96.7%) and a higher predictive power than ApoA1 and ApoB alone (Fig. 3c and Supplementary Fig. 5a-b). From this analysis a sensitive and specific cut-off value for ApoB:ApoA1 ratio of 0.45 was established to enable these groups to be distinguished from one another (Fig. 3d). The ApoB:ApoA1 ratio was also statistically significantly elevated in Group-1/1A compared to the unvalidated Group-3 and was statistically significantly higher than age matched adolescent healthy donors (Group-1/1A, n=30, mean= 0.64, CI= 0.56-0.71; HC, n=32, mean= 0.47, CI=0.42-0.51; p=0.0001, t test) (Supplementary Fig. 6a).

Serum ApoB:ApoA1 ratio is known to be a more effective CVR predictor than routine cholesterol measurements (a higher ratio is associated with increased CVR)[23,24]. In support of this, our results showed that routine clinical measures of lipids and atherosclerosis were less effective at distinguishing between the groups (Supplementary Fig. 6b). Furthermore, metabolites associated with pre-clinical carotid and femoral artery atherosclerotic plaque in adult-onset SLE patients [35] correlated statistically significantly with the ApoB:ApoA1 ratio in patients from Group-1/1A only (Supplementary Fig. 6c). We observed no statistically significant difference in ApoB:ApoA1 between males and females with JSLE (Supplementary Fig. 6d), despite differences in sex ratios across cohorts (Table 1). Finally, longitudinal analysis of ApoB:ApoA1 ratios from multiple patient visits over a 12 month period showed that the ApoB:ApoA1 ratio biomarker remained stable over time (Fig. 3e). Thus elevated ApoB:ApoA1 ratio could be a potential biomarker of increased CVD risk in adolescent JSLE patients.

### 3.3 JSLE patients with high ApoB:ApoA1 ratio have a unique T-cell phenotype and transcriptional profile

**Fig. 4:**
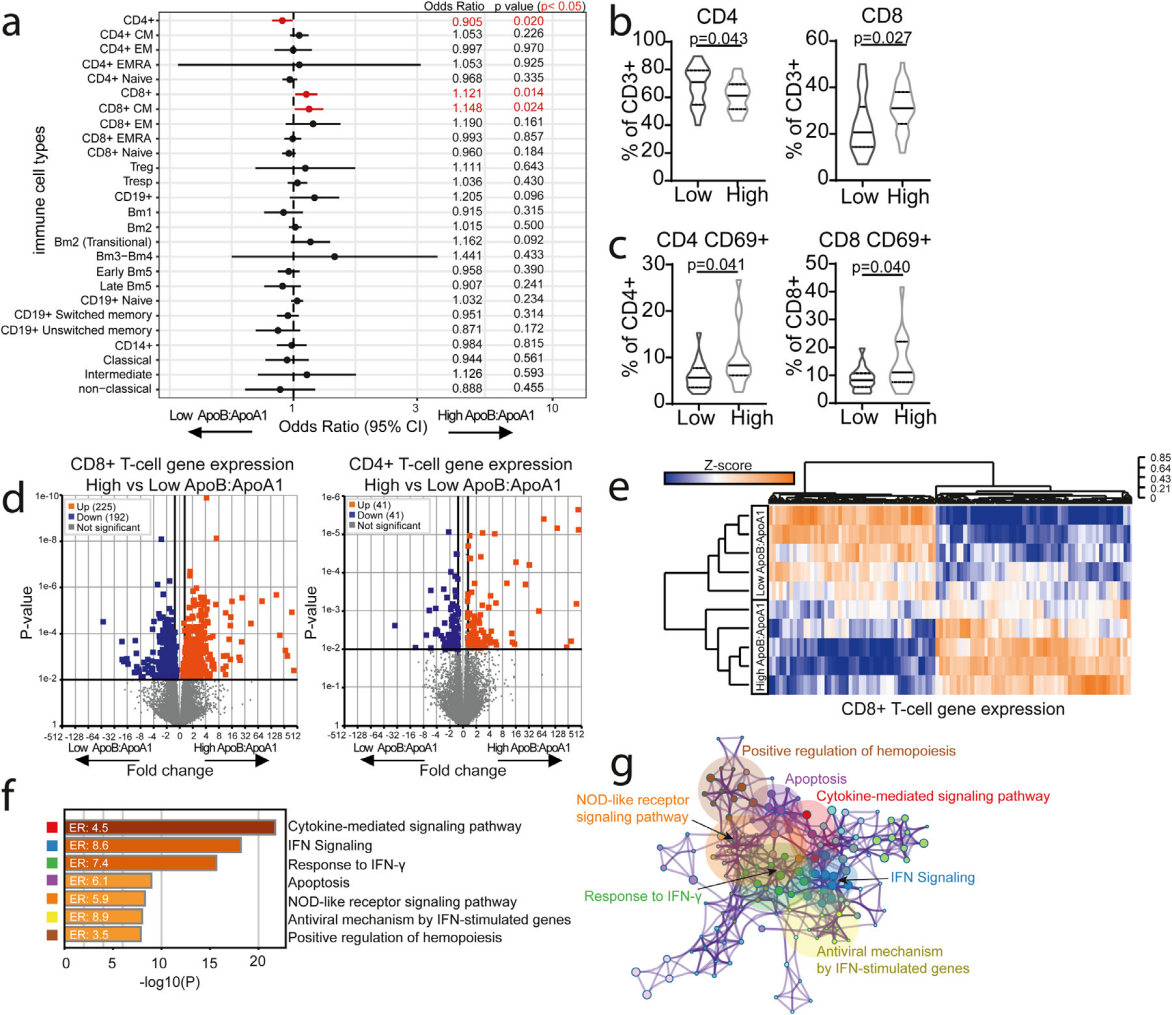
*High ApoB:ApoA1 ratio patients have a unique T-cell phenotype and transcriptomic signature***.** Peripheral blood mononuclear cells (PBMC's) from high (n=29) and low (n=25) ApoB:ApoA1 ratio JSLE patients were stained ex-vivo to evaluate expression of 28 immune cell subsets by flow cytometry (Supplementary Fig. 1 for gating strategy). **(a)** Forest plot showing ORs and 95% CIs of 28 immunological parameters analysed by logistic regression adjusted for BMI, sex, age, ethnicity, disease duration, treatment, SLEDAI-2000, CRP, dsDNA, C3. Dotted line represents no effect (OR=1), statistically significant differences between high and low ApoB:ApoA1 patients (in red). iNKT and PDC data is not shown due to very different CI values. See Supplementary Data Table 2 for CIs. **(b-c)** Violin plots displaying CD4+ and CD8+ T-cell frequencies **(b)** and CD69 expression **(c)** between high and low ApoB:ApoA1 ratio groups. T test. FACS-sorted CD8+ (n=5/group) and CD4+ (n=6/group) T-cells from JSLE patients with high and low ApoB:ApoA1 ratio were analysed by RNA-sequencing and whole genome expression compared between the groups. **(d)** Volcano plots displaying fold changes and p-values, where coloured points represent statistically significantly regulated genes. (**e-g**) RNA-sequencing analysis for CD8+ T cells. (**e**) Clustered heatmap of normalised gene counts of statistically significantly altered genes with adjusted p-value threshold (<0.01) **(f**) Bar charts plotting cluster significance and enrichment ratio (ER) of enriched pathway ontology terms between high and low ApoB:ApoA1 JSLE patients. **(g)** Network diagram illustrating statistically significantly enriched genetic pathway ontology terms between high and low ApoB:ApoA1 JSLE patients. Similar terms with a high degree of redundancy were clustered into groups as depicted. Each node represents a statistically significantly enriched term, with node size proportional to the number of input genes annotated with this term. See Supplementary Fig. 8.

Inflammation is known to accelerate atherosclerosis development in patients with JSLE [42], therefore the immune cell profile of JSLE patients in the High vs Low ApoB:ApoA1 groups was evaluated by logistic regression analysis (adjusted for BMI, sex, age, ethnicity, disease duration, treatment, SLEDAI-2000, hsCRP, dsDNA, and C3) in matched patient samples to the baseline metabolomics. Differences between High and Low ApoB:ApoA1 groups were exclusive to the T-cell compartment; specifically, we identified statistically significantly decreased total CD4+ T-cells, increased total and central memory (CM)CD8+ T-cells and increased CD4+ and CD8+ T-cell activation in the High ApoB:ApoA1 group (Fig. 4a-c, Supplementary data Table 2). Of note, these individual immune cell subsets had a reduced ability to differentiate between the two patient groups compared to ApoB:ApoA1 ratio (Supplementary Fig. 7).

The differential T-cell profile was further explored using transcriptomic analysis in a subset of matched patient samples. Differentially expressed genes (DEGs) were identified in both CD8+ (225 up- and 192 down-regulated) and CD4+ T-cells (41 up- and 41 downregulated) (Fig. 4d and Supplementary Table 7) from JSLE patients in the High versus Low ApoB:ApoA1 groups. Statistically significantly enriched pathways were clustered into biologically relevant groups which were principally associated with response to IFN, cytokine-mediated signalling, and apoptosis in CD8+ T-cells (Fig. 4e-g) and response to oxidative stress, gonadotropin-releasing hormone (GnRH) signalling and the nucleotide biosynthetic process in CD4+ T-cells (Supplementary Fig. 8a-d). Interestingly, 34 CD8+ and 2 CD4+ T-cell genes overlapped with DEGs in T-cells isolated from human atherosclerotic plaque[43] (Supplementary Fig. 8e, Supplementary Table 8), suggesting that CD8+ T-cells in particular, may play an important role the immune-mediated response to a pro-atherogenic environment in patients with JSLE.

### 3.4 Common IFN signalling pathways are regulated in JSLE patients with High ApoB:ApoA1 and human atherosclerotic plaques

**Fig. 5:**
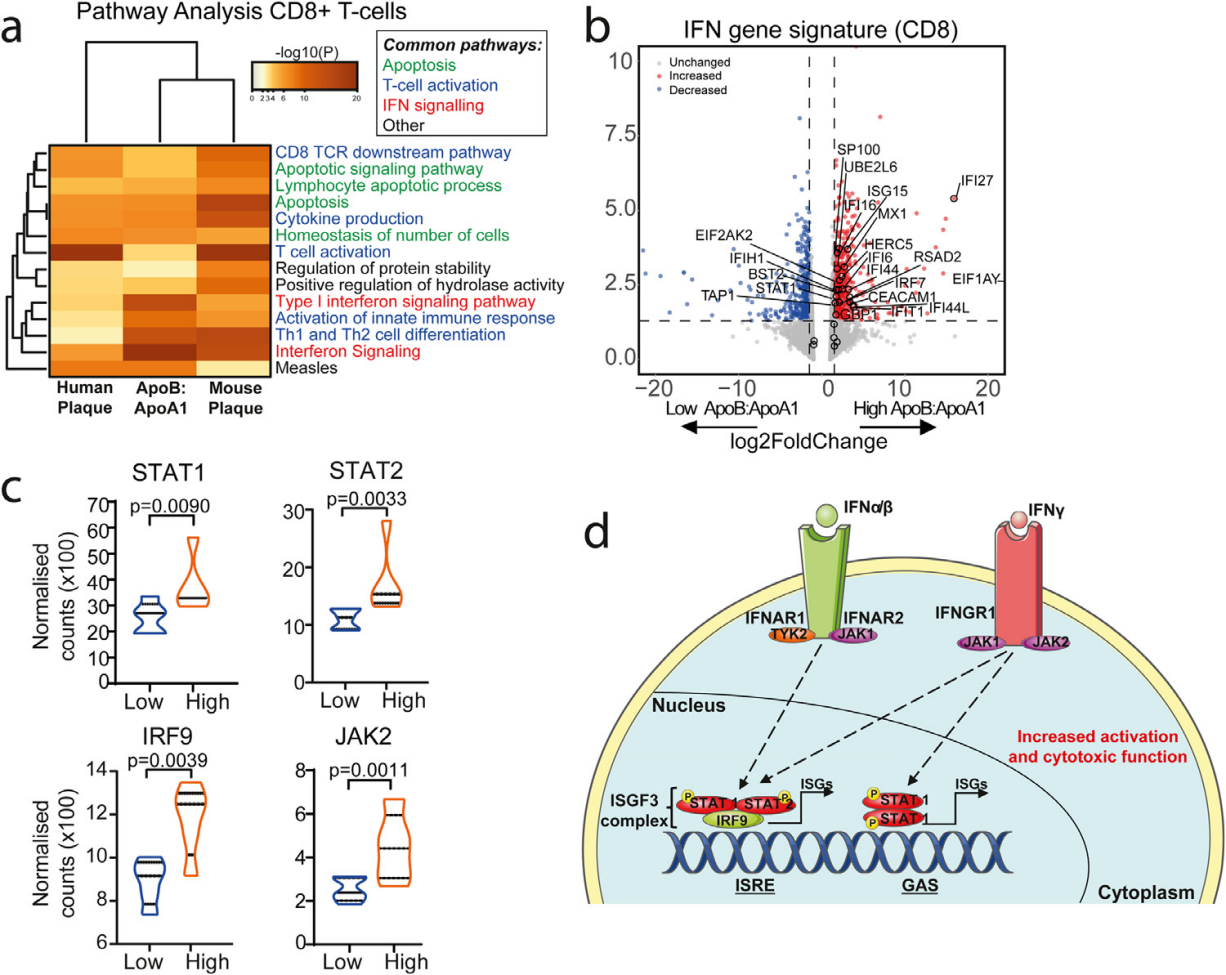
*High ApoB:ApoA1 ratio patients have a CD8+ T-cell transcriptomic profile associated with atherosclerosis and interferon signalling.***(a)** Heatmap displaying -log10 p values of statistically significantly enriched genetic pathway ontology terms that overlap in CD8+ T-cell gene list analysis from human atherosclerotic plaques [43] (left), JSLE patients with high vs low ApoB:ApoA1 ratio (n=5/group) (middle), and mouse atherosclerotic plaques [44] (right). **(b)** Volcano plot (VolcaNoseR https://huygens.science.uva.nl/VolcaNoseR, date last accessed July 2020) showing genes associated with the Interferon (IFN) gene pathway ontology term identified in (a) in CD8+ T-cells from JSLE patients with high vs low ApoB:ApoA1 ratio (n=5/group). Genes overlapping with the SLE-associated IFN signature described in [45] are labelled. Log2 Fold change and p-value plotted, coloured points represent statistically significantly regulated genes. **(c)** Violin plots comparing normalised CD8+ T-cell gene counts from JSLE patients with high vs low ApoB:ApoA1 (n=5/group) in the IFN-stimulated gene factor 3 transcription complex (ISGF3) complex IFN signaling pathway. Unpaired t test. **(d)** Summary diagram of ISGF3 signaling. Abbreviations: Interferon-α/β/γ (IFNα/β/γ), interferon regulatory factor 9 (IRF9), interferon stimulated genes (ISGs). Janus kinase 2 (JAK2), signal transducer and activator of transcription 1/2 (STAT1/2), tyrosine kinase 2 (TYK2), interferon alpha and beta receptor subunit 1/2 (IFNAR1/2), interferon gamma receptor 1 (IFNGR1), interferon sensitive response element (ISRE), gamma-activated sequence (GAS), phosphate group (P) (also see Supplementary Fig. 9).

Pathway enrichment analysis of DEGs identified multiple commonly regulated pathways in CD8+ T-cells from JSLE patients with High vs Low ApoB:ApoA1 compared with DEGs in CD8+ T-cells isolated from human [43] and mouse [44] atherosclerotic plaques (available from previously published work) (Fig. 5a). These pathways included those associated with apoptosis, T-cell activation and IFN signalling (Fig. 5a, Supplementary Fig. 9a-d and Supplementary Table 9). The IFN signalling pathway was statistically significantly upregulated in CD8+ T-cells from JSLE patients with High ApoB:ApoA1, including 25 out of 31 genes from a recently identified SLE-associated IFN signature [45] (Fig. 5b). Nine of these genes were identified in the pathway analysis of all three CD8+ T-cell datasets: BST2, IFI6, ISG15, MX1, RSAD2, SP100, STAT1, UBE2L6 and HERC5 (Supplementary Fig. 9e), suggesting that these SLE-associated IFN genes could also be associated with atherogenesis. Notably, genes associated with the IFN-stimulated gene factor 3 transcription complex (ISGF3) pathway were upregulated in JSLE patients with High ApoB:ApoA1 including STAT1, STAT2, IRF9 and JAK2 (Fig. 5c-d). STAT-1 and IRF9 were also significantly increased in mouse atherosclerotic plaque [44]. None of these genes were statistically significantly dysregulated in CD4+ T-cells (Supplementary Fig. 9f, Supplementary Table 7). Thus, these data suggest that there could be a relationship between circulating atherogenic lipids, IFN signalling and activation in CD8+ T-cells, providing a potential mechanistic insight into the pathogenesis of atherosclerosis in JSLE.

### 3.5 JSLE patients with High ApoB:ApoA1 ratio have a more active longitudinal disease trajectory

**Fig. 6:**
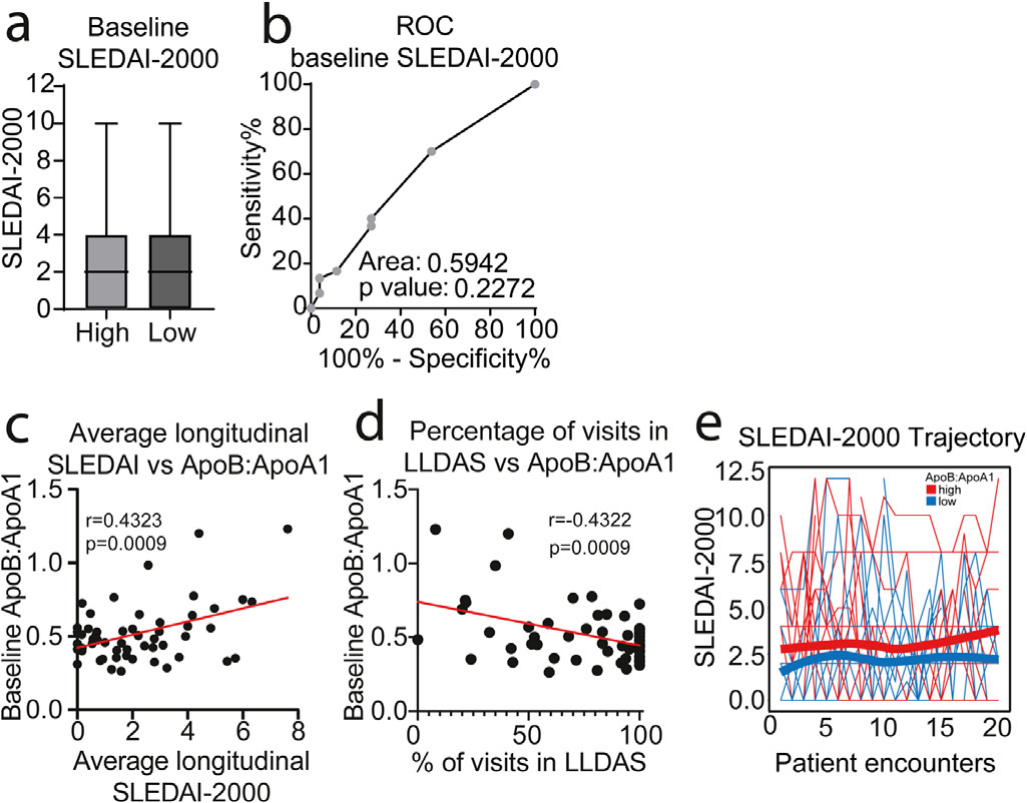
*Longitudinal analysis of disease activity differs between high and low ApoB:ApoA1 ratio groups***. (a)** Systemic Lupus Erythematosus Disease Activity index (SLEDAI-2000) comparison between high (n=30, red) and low (n=26, blue) ApoB:ApoA1 ratio JSLE patients. Box and Whisker plot showing mean and 1st/3rd quartiles and minimum/maximum values, T test. **(b)** ROC curve analysis of baseline SLEDAI-2000 score of patients in Group-1/1A (n=30) compared to patients in Group-2/2A (n=26). Area under the curve (AUC) displayed. **(c)** Pearson's correlation between baseline ApoB:ApoA1 ratio and average SLEDAI-2000 of JSLE patients (n=56) measured over 3-7 years of follow up (mean years of follow-up per patient=4.9, mean number of visits per patient=17.1). **(d)** Pearson's correlation between baseline ApoB:ApoA1 ratio and longitudinal Lupus Low Disease Activity State (LLDAS) (n=56). **(e)** Longitudinal assessment of JSLE patient (n=56) SLEDAI-2000 score trajectories over 20 clinical encounters displayed as spaghetti plots. Each line represents one JSLE patient. Red=patients with high baseline ApoB:ApoA1; Blue=patients with low baseline ApoB:ApoA1. Smoothing lines were added to indicate the trend of high and low ApoB:ApoA1 groups.

Disease activity has been associated with lipid abnormalities in JSLE patients[9]. At baseline (the time point for all metabolomics, immune cell phenotype and whole gene expression analysis), there was no statistically significant difference in disease activity (SLEDAI-2000) between ApoB:ApoA1 patient groups (Fig. 6a-b and Supplementary Tables 3 and 4). However, follow up of disease activity over 3-7 years (mean=4.9 years) revealed that the baseline ApoB:ApoA1 ratio correlated positively with average longitudinal SLEDAI-2000 score and negatively with longitudinal Lupus Low Disease Activity State (LLDAS) (Fig. 6c-d). Furthermore, despite the majority of JSLE patients having low disease activity, trajectory analysis of SLEDAI-2000 score (over 20 individual patient follow up encounters) showed a small but consistently more active disease profile in patients with High ApoB:ApoA1 ratio (Fig. 6e). Thus elevated ApoB:ApoA1 ratio was associated with a trend towards a more active disease trajectory over time.

Together, the results suggest that ApoB:ApoA1 ratio could be a robust biomarker for prediction of increased CVD risk in patients with JSLE, even in patients with relatively low disease activity. These patients with potentially high CVD risk could not be distinguished using routine clinical assessment or standard lipid measures and had a unique immune cell and transcriptomic profile. Lipid modification therapies and/or changes in diet could be beneficial to modify the CVD risk and inflammatory profile in patients with a high ApoB:ApoA1 ratio (Supplementary Fig. 10).

## 4 Discussion

Patients with JSLE have an increased risk of developing CVD. Despite this knowledge, CVD is still the leading comorbidity for patients, highlighting the need for better understanding of the disease process [2,5]. Using metabolomics analysis this study identified ApoB:ApoA1 ratio as a potential predictive biomarker of increased cardiometabolic risk in adolescent patients with JSLE. Patients with high ApoB:ApoA1 ratio shared a serum metabolomic profile with adult SLE patients with sub-clinical atherosclerosis; a CD8+ T-cell phenotype with CD8+ T-cells isolated from human atherosclerotic plaque; and had a more active disease trajectory. Based on these findings we propose that detailed lipoprotein taxonomy analysis could help distinguish JSLE patients with potentially increased CVD risk. This work could both improve clinical trial design and identify patients that could benefit from more tailored disease or lipid-modifying therapy.

Prediction of CVD in the general population predominantly relies on relatively simple measurements such as serum total cholesterol, HDL, LDL and triglycerides [46]. However, more in-depth lipoprotein assessment including lipoprotein subclasses, their lipid concentrations and composition can provide new insights into CVD risk prediction and disease mechanisms [37,47,48]. This approach could be particularly relevant for assessing CVD risk in younger patients with chronic inflammatory conditions such as JSLE, for whom the relevance of traditional risk factors in CVD prediction is less robust [3].

Elevated ApoB:ApoA1 ratio is associated with increased CVD risk in young people. The Cardiovascular Risk in Young Finns Study shows that elevated ApoB and ApoB:ApoA1 ratio and reduced ApoA1 in children and adolescents reflects a predisposition to subclinical atherosclerosis in adulthood in healthy individuals [49]. In support of our study other reports show that ApoB:ApoA1 ratio has an improved CVD predictive value compared to conventional blood cholesterol measures [23,24]; this could be due to the exclusion of VLDL and IDL in the majority of these measures. Our data suggests that converting conventional blood cholesterol measures into ratios could also provide promising biomarkers of CVR in JSLE as they also discriminated between the stratified groups with high performance and have the additional practicality benefit over NMR. There is evidence that ApoA1 and ApoB could be measured in clinical practice facilitated by commercially available colorimetric assays; however, further research is required to compare their performance against NMR spectroscopy. JSLE patients can have reduced ApoA1 and HDL concentration compared with healthy donors [50] and adult SLE patients have increased Apo‐B associated with arterial stiffness [51]. Importantly, we show here that in adolescent JSLE patients, ApoB:ApoA1 ratio statistically significantly correlates with metabolites associated with sub-clinical carotid and/or femoral atherosclerotic plaque in adult SLE patients [35]. We identified a cut-point value for ApoB:ApoA1 ratio of 0.45 that could have clinical utility in JSLE. However, ApoB:ApoA1 ratio is age dependent so further validation of age specific cut points is needed [37,52,53]. Differences in published ratios could be accounted for by different technologies used to assess ApoB and ApoA1 levels (mainly colorimetry-based compared to NMR used here) and the fasting status of the study participants (our study was performed on non-fasting patients). We expect that any longitudinal fluctuations in the ApoB:ApoA1 ratio were due to patient flares, rather than changes in diet or BMI, as this is unlikely to change significantly at 3-month intervals. Importantly, although we adjusted for sex in the metabolomic logistic regression analysis, we identified no statistically significant difference in ApoB:ApoA1 ratio between males and females with JSLE, despite previous reports of sex differences in lipid profile in healthy individuals [54], reflecting the known increased CVR in young women with SLE. Several studies have shown that sub-clinical atherosclerosis in SLE begins in the rare paediatric age group and highlight the need for paediatric rheumatologists to address modifiable CVR factors in daily practice [6,20]. However, not all patients with JSLE have the same CVD risk; therefore, identifying patients with a potential low and high CVD risk could be pivotal for the success of anti-CVD interventions. For example, while the APPLE trial of atorvastatin in JSLE patients did not show reduced progression of sub-clinical atherosclerosis overall, a trend towards slower carotid intima-media thickness (CIMT) progression was seen [20]. The authors suggested certain subgroups of JSLE patients would benefit from targeted statin therapy. Thus, stratifying JSLE patients according to ApoB:ApoA1 ratio could help future clinical trial design to assess efficacy of therapies targeting CVD risk.

JSLE patients with elevated ApoB:ApoA1 ratio were characterised by a distinct CD8+ T-cell immune profile. There was an overlap between CD8+ T-cell DEGs from patients with High ApoB:ApoA1 ratio with those identified in individuals presenting with symptomatic atherosclerotic plaques [43], helping us to understand the processes involved in the association between High ApoB:ApoA1 ratio and increased CVD risk. CD8+ T-cells are the most abundant cell type in human atherosclerotic plaques (>30%) according to recent single-cell RNA sequencing data, with increased frequency in plaque compared to blood [43]. However, other studies focusing on CD8+ T-cells in atherosclerosis are limited and conflicting, describing plaque-derived T-cells as either pro- or anti-atherogenic [55], [56], [57], [58]. Thus, greater understanding of the immune processes leading to human atherosclerotic plaque-development are needed, in particular in the context of chronic inflammation and immune cell dysregulation, which may speed atherosclerosis progression in patients with autoimmune diseases [42,59,60].

Notably altered IFN signalling gene expression pathways were shared between peripheral blood CD8+ T-cells from High ApoB:ApoA1 JSLE patients and CD8+ T-cells from symptomatic human plaque. Both Type-I and Type-II IFNs can contribute to atherosclerosis progression [61,62]. Furthermore, both SLE pathogenesis and SLE sub-clinical CVD are associated with Type-I IFNs [45,[63], [64], [65]]. The IFN-stimulated gene factor 3 (ISGF3) complex was statistically significantly upregulated in CD8+ T-cells from the High ApoB:ApoA1 group. Activation of this signalling pathway, including phosphorylation of signal transducers and activators of transcription (STAT)1 and STAT2 and heterodimerization with IFN regulatory factor (IRF)9 (part of the ISGF3 complex) can lead to pro-inflammatory and atherogenic responses [66], [67], [68]. In support of this, an antimalarial drug, artesunate, downregulated IFN induced STAT1 phosphorylation in SLE PBMC's *in vitro*, which reduced production of macrophage migration inhibitory factor (MIF), a key regulator of both atherosclerosis and SLE [69]. Clinical trials blocking Type-I IFN and JAK/STAT signalling are underway and could help target atherosclerosis as well as autoimmune inflammation [70,71]. In addition, patients with a high ApoB:ApoA1 ratio had increased longitudinal disease activity over 5 years follow up. Interplay between altered circulating lipids and the pro-inflammatory profile of CD8^+^ T-cells could partly explain these outcomes, as suggested by increased IFN signalling pathways, further supporting the use of these anti-inflammatory therapies. It is important to note that CD4^+^ T-cells in patients with a high ApoB:ApoA1 ratio showed increased response to oxidative stress signalling, a process that has been implicated in atherosclerosis through oxidization of LDL and altered inflammatory processes [72].

This project has some limitations. Our cohort included mainly patients with low disease activity; therefore, it will be important in future studies to investigate further the lipidomic profile of active and inactive JSLE patients and their utility versus conventional lipid measurements. We are aware of the impact of drugs including hydroxychloroquine, rituximab and cyclophosphamide on lipid profiles [15,29] and patients treated with rituximab and cyclophosphamide within the past year were excluded from the study. There can be challenges related to oral treatment compliance in this patient population, we did not measure blood levels of hydroxychloroquine or steroids to check compliance to treatment dose. None of the JSLE patients included in our study declared that they were smokers; but again we recognise the limitations of self-reported data, especially in this age group. Measurement of endothelial or vascular dysfunction and assessment for presence of atherosclerotic plaque in this patient group was beyond the scope of the study; we used an adult SLE patient group scanned for presence of subclinical atherosclerotic plaque to validate the identified lipid biomarkers and used published literature data to validate DEGs from CD8+ T-cells isolated from human/mouse atherosclerotic plaques. The small sample size used in this study may be masking further clinically important results. It will be important to validate this work in a larger multi-centre study to increase the sample size for metabolomic and transcriptomic analyses, as well as account for genetic, environmental and dietary differences in JSLE, although this cohort was ethnically diverse and we accounted for the majority of conventional CVD risk factors and disease outcome measures in our analysis. JSLE is a rare disease, representing only 15-20% of all SLE patients, thus recruitment of large patient numbers within this age group poses numerous challenges. Future research will address both clinical validation (including endothelial dysfunction studies and measurement of plaque and intimal-media thickness) as well as potential therapeutic interventions.

In conclusion, this study, identified relationships between altered lipid taxonomy, immune cell profiles and gene expression patterns in JSLE patients, which could be associated with increased cardiometabolic risk. There are no guidelines for CVD risk monitoring or management in patients with SLE/JSLE, thus there is an urgent need to find better ways to stratify these patients based on their CVD risk and identify adequate therapeutic approaches to decrease the overall CVD morbidity and mortality associated with SLE. This study proposes high ApoB:ApoA1 ratio as a potential biomarker to stratify JSLE patients for a more personalised approach to lipid modification therapy.

## Contributors

Design of research study; ECJ, IPT, CC, YI. Acquiring data; GAR, KEW, MN, AR, HP, CW. Recruiting patients; CC, YI, CW, DAI. Analyzing data; GAR, LC, JP, KEW, ECJ, IPT: Writing the manuscript; GAR, ECJ: Review of the manuscript; CC, IPT, DAI. All authors reviewed and edited the final version.

## Declaration of Competing Interest

The authors have declared that no conflict of interest exists.

## Appendix Supplementary materials

Image, application 1
