# Supplementary material for: Increased apolipoprotein-B:A1 ratio predicts cardiometabolic risk in patients with juvenile onset SLE
Source: eBioMedicine. 2021 Feb 24;65:103243. doi: 10.1016/j.ebiom.2021.103243 (PMC7992074; doi:10.1016/j.ebiom.2021.103243)
Supplement: Supplementary file 1 [file mmc1.docx]

**Supplemental Material**

Increased Apolipoprotein-B:A1 ratio predicts cardiovascular risk in patients with juvenile onset SLE

**Patient cohort.** Study protocol excerpt; eligibility recruitment and sample size. *Page 2*

**Supplementary Table 1.** List of Metabolic biomarkers. *Page 3*

**Supplementary Figure 1.** Representative flow cytometry gating strategies. *Page 5*

**Supplementary Figure 2.** Study design and analysis plan flow diagram. *Page 6*

**Supplementary Table 2.** Metabolomics in Discovery JSLE cohort: Groups-1, -2 and -3. *Page 7*

**Supplementary Figure 3.** Patients in Gp1/G1A and Gp2/2A cluster together in a PCA analysis.

*Page 8*

**Supplementary Table 3.** Demographic and treatment information in the stratified JSLE patient groups (Discovery cohort). *Page 9*

**Supplementary Table 4.** Demographic and treatment information in the two JSLE validation cohort groups. *Page 11*

**Supplementary Figure 4.** Odds ratios of non-lipid metabolites between high and low ApoB:ApoA1 ratio groups adjusting for clinical parameters. *Page 13*

**Supplementary Table 5.** Significant metabolites distinguishing high and low ApoB:ApoA1 ratio groups following adjustment for all clinical data, treatment and BMI. *Page 14*

**Supplementary Table 6.** Principle component analysis factor loadings of the significant metabolites distinguishing high and low ApoB:ApoA1 ratio groups. *Page 14*

**Supplementary Figure 5.** ROC curve analysis of top differentially expressed metabolites between Group-1/1A and Group-2/2A. *Page 15*

**Supplementary Figure 6.** ApoB:ApoA1 ratio correlates with biomarkers associated with pre-clinical plaque in Group-1/1A but not Group-2/2A or 3. *Page 16*

**Supplementary Figure 7.** ROC curve analysis of significantly altered immune cell subsets between Group-1/1A and Group-2/2A. *Page 16*

**Supplementary Table 7.** List of DEGs in CD4+ and CD8+ T-cells from JSLE patients with High vs Low ApoB:ApoA1 ratios. *Page 18*

**Supplementary Figure 8:** DEGs in T-cells from JSLE patients with High vs Low ApoB:ApoA1 ratios. *Page 20*

**Supplementary Table 8.** List of overlapping DEGs between T-cells from JSLE patients with High vs Low ApoB:ApoA1 ratios and human atherosclerotic plaque.  *Page 21*

**Supplementary Figure 9.** Genes associated with apoptosis, T-cell activation and interferon signalling significantly altered between JSLE patients with high vs low ApoB:ApoA1 ratio*. . Page 23*

**Supplementary Table 9.** List of genes associated with overlapping CD8+ T-cell pathways *Page 24*

**Supplementary Figure 10.** Phenotypic summary of the stratified JSLE patient groups.  *Page 27*

**Data Table**

**Data Table 1.** Normalised logistic regression metabolomics data comparing Group-1/1A and Group-2/2A *Page 29*

**Data Table 2:** Logistic regression immune phenotype data comparing Group-1/1A and Group-2/2A

*Page 33*

**Patient cohort**

**Study protocol excerpt: Extracted from Adolescent Centre for Rheumatology – Centre Ethics protocol document (**REC no. Ref.11/LO/0330).

**Eligibility and recruitment:**

Inclusion:

- An autoimmune rheumatic disease fulfilling internationally recognised consensus classification criteria. Patients with juvenile systemic erythematosus (JSLE) should fulfil the 1997 American College of Rheumatology (ACR)(1) or/and the 2012 Systemic Lupus International Collaborating Clinics (SLICC)(2) classification criteria, and be diagnosed before 18 years of age.
- Aged 6 years or older
- Puberty tanner stage 4-5

Exclusion:

- Any patient who withholds consent or whose carer withholds consent (as appropriate given patient’s competence)
- Any patient who withdraws from the study

**Additional criteria for this paper:**

Inclusion: JSLE samples with more than 10 million PBMCs/sample.

Exclusion: patients treated with Rituximab or Cyclophosphamide in the last 12 months

**Sample size:**

JSLE is a rare disease; this was an exploratory study based on the number of patients and healthy donors available fitting the inclusion/exclusion criteria. The discovery (n=31) and validation (n=31) cohorts were split based on time of eligible door blood collection in an unbiased manor. For baseline analysis, patients were included at one time point only, however, for a select few patients, longitudinal blood samples were collected. The data provided here will provide a sound basis for future work.

**Demographic, Clinical and Treatment data collected at baseline and longitudinal follow-up:**

Demographics (age, sex, ethnicity, BMI, disease duration); Serology (dsDNA, extractable nuclear antigens, CRP, C3, ESR, lymphocyte count, anti-cardiolipin antibodies, clinical lipid measures, urine protein:creatinine ratio); Organ involvement (renal, central nervous system, musculoskeletal, haematological, skin); Co-morbidities; Disease activity scores SLE Disease Activity Index-2000 (SLEDAI-2000)(3) and Lupus Low Disease Activity State (LLDAS) (4); Treatment (Hydroxychloroquine, Mycophenolate mofetil, Prednisolone, Vitamin D, Methotrexate, Azathioprine, Rituximab, Cyclophosphamide). No patients had diabetes. All patients had well controlled BP and no patient had consistently increased BP above 135/85.

**Supplementary Table 1. List of Metabolic biomarkers**

| - **Cholesterol** | - **Lipoprotein subclasses** | - M-LDL-CE |
| --- | --- | --- |
| - Total-C | - XXL-VLDL-P | - M-LDL-FC |
| - VLDL-C | - XXL-VLDL-L | - M-LDL-TG |
| - Remnant-C | - XXL-VLDL-PL | - S-LDL-P |
| - LDL-C | - XXL-VLDL-C | - S-LDL-L |
| - HDL-C | - XXL-VLDL-CE | - S-LDL-PL |
| - HDL2-C | - XXL-VLDL-FC | - S-LDL-C |
| - HDL3-C | - XXL-VLDL-TG | - S-LDL-CE |
| - Esterified-C | - XL-VLDL-P | - S-LDL-FC |
| - Free-C | - XL-VLDL-L | - S-LDL-TG |
| - **Glycerides and phospholipids** | - XL-VLDL-PL | - XL-HDL-P |
| - Total triglycerides | - XL-VLDL-C | - XL-HDL-L |
| - VLDL-TG | - XL-VLDL-CE | - XL-HDL-PL |
| - LDL-TG | - XL-VLDL-FC | - XL-HDL-C |
| - HDL-TG | - XL-VLDL-TG | - XL-HDL-CE |
| - Phosphoglycerides | - L-VLDL-P | - XL-HDL-FC |
| - TG/PG | - L-VLDL-L | - XL-HDL-TG |
| - Total cholines | - L-VLDL-PL | - L-HDL-P |
| - Phosphatidylcholines | - L-VLDL-C | - L-HDL-L |
| - Sphingomyelins | - L-VLDL-CE | - L-HDL-PL |
| - **Apolipoproteins** | - L-VLDL-FC | - L-HDL-C |
| - ApoB | - L-VLDL-TG | - L-HDL-CE |
| - ApoA1 | - M-VLDL-P | - L-HDL-FC |
| - ApoB/ApoA1 | - M-VLDL-L | - L-HDL-TG |
| - **Fatty acids** | - M-VLDL-PL | - M-HDL-P |
| - FAw3/FA | - M-VLDL-C | - M-HDL-L |
| - FAw6/FA | - M-VLDL-CE | - M-HDL-PL |
| - PUFA/FA | - M-VLDL-FC | - M-HDL-C |
| - MUFA/FA | - M-VLDL-TG | - M-HDL-CE |
| - SFA/FA | - S-VLDL-P | - M-HDL-FC |
| - DHA/FA | - S-VLDL-L | - M-HDL-TG |
| - LA/FA | - S-VLDL-PL | - S-HDL-P |
| - **Amino acids** | - S-VLDL-C | - S-HDL-L |
| - Alanine | - S-VLDL-CE | - S-HDL-PL |
| - Glutamine | - S-VLDL-FC | - S-HDL-C |
| - Glycine | - S-VLDL-TG | - S-HDL-CE |
| - Histidine | - XS-VLDL-P | - S-HDL-FC |
| - Isoleucine | - XS-VLDL-L | - S-HDL-TG |
| - Leucine | - XS-VLDL-PL |  |
| - Valine | - XS-VLDL-C |  |
| - Phenylalanine | - XS-VLDL-CE |  |
| - Tyrosine | - XS-VLDL-FC |  |
| - **Glycolysis related metabolites** | - XS-VLDL-TG |  |
| - Glucose | - IDL-P |  |
| - Lactate | - IDL-L |  |
| - Pyruvate | - IDL-PL |  |
| - Citrate | - IDL-C |  |
| - Glycerol | - IDL-CE |  |
| - Ketone bodies | - IDL-FC |  |
| - Acetate | - IDL-TG |  |
| - Acetoacetate | - L-LDL-P |  |
| - 3-hydroxybutyrate | - L-LDL-L |  |
| - **Fluid balance** | - L-LDL-PL |  |
| - Creatinine | - L-LDL-C |  |
| - Albumin | - L-LDL-CE |  |
| - **Inflammation** | - L-LDL-FC |  |
| - Glycoprotein acetyls | - L-LDL-TG |  |
| - **Lipoprotein particle sizes** | - M-LDL-P |  |
| - VLDL particle size | - M-LDL-L |  |
| - LDL particle size | - M-LDL-PL |  |
| - HDL particle size | - M-LDL-C |  |

**Supplementary Table 1. List of Metabolic biomarkers**

Nightingale Health metabolomics platform (https://nightingalehealth.com/) was used to measure biomarkers in JSLE patient serum. This service measures blood metabolic biomarkers using nuclear magnetic resonance (NMR) spectroscopy. The platform can simultaneously measure amino acids, fatty acids, glycolysis metabolites, routine lipid measures (mmol/l), apolipoproteins (g/l) and in depth lipoprotein measurements such as particle size (nm), concentration and lipid content (mmol/l). The platform provides repeatable measurements with no batch effects. The Nightingale Health service has been thoroughly validated and has been used to measure over 500,000 samples from both research and clinical trials.

Abbreviations: Apo, apolipoprotein; VLDL, very low density lipoprotein; IDL, intermediate density lipoprotein; LDL, low density lipoprotein; HDL, high density lipoprotein; XXL-VLDL, chylomicrons and extremely large VLDL; X-Large, very large; X-small, very small; Est (esterified), PG (Phosphoglyceride), PC, Phosphatidylcholine; SM, Sphingomyelins; Unsat, Unsaturated; DHA, Docosahexaenoic acid; LA, Linoleic acid; FAw3, Omega-3 fatty acids; FAw6, Omega-6 fatty acids; PUFA, Polyunsaturated fatty acids; MUFA, Monounsaturated fatty acids; SFA, Saturated fatty acids; TG, triglycerides; PL, phospohlipids, FC, free cholesterol, C, cholesterol, CE, cholesterol esters; P, particle; L, lipid.

**Supplementary Figure 1. Representative flow cytometry gating strategies**

**

**

**Supplementary Figure 1. Representative flow cytometry gating strategies.** Representative gating strategies from a healthy donor used to identify **(a)** live and single cell **(b)** T-cell, **(c)** Monocyte and PDC and **(d)** B-cell subsets. PBMC’s were stained with fluorescently labelled antibodies and measured by flow cytometry. Labels represent the cell population within the gate and the percentage of parent gate. Abbreviations: Regulatory T-cells (Tregs), invariant natural killer T-cells (iNKT-cells), central memory (CM), effector memory (EM), plasmacytoid dendritic cell (PDC), Bm1 (naïve), Bm2 (mature), Bm2’ (transitional), Bm3-4 (Plasmablasts), early/late Bm5 (memory).

**Supplementary Figure 2. Study design and analysis plan flow diagram**

**
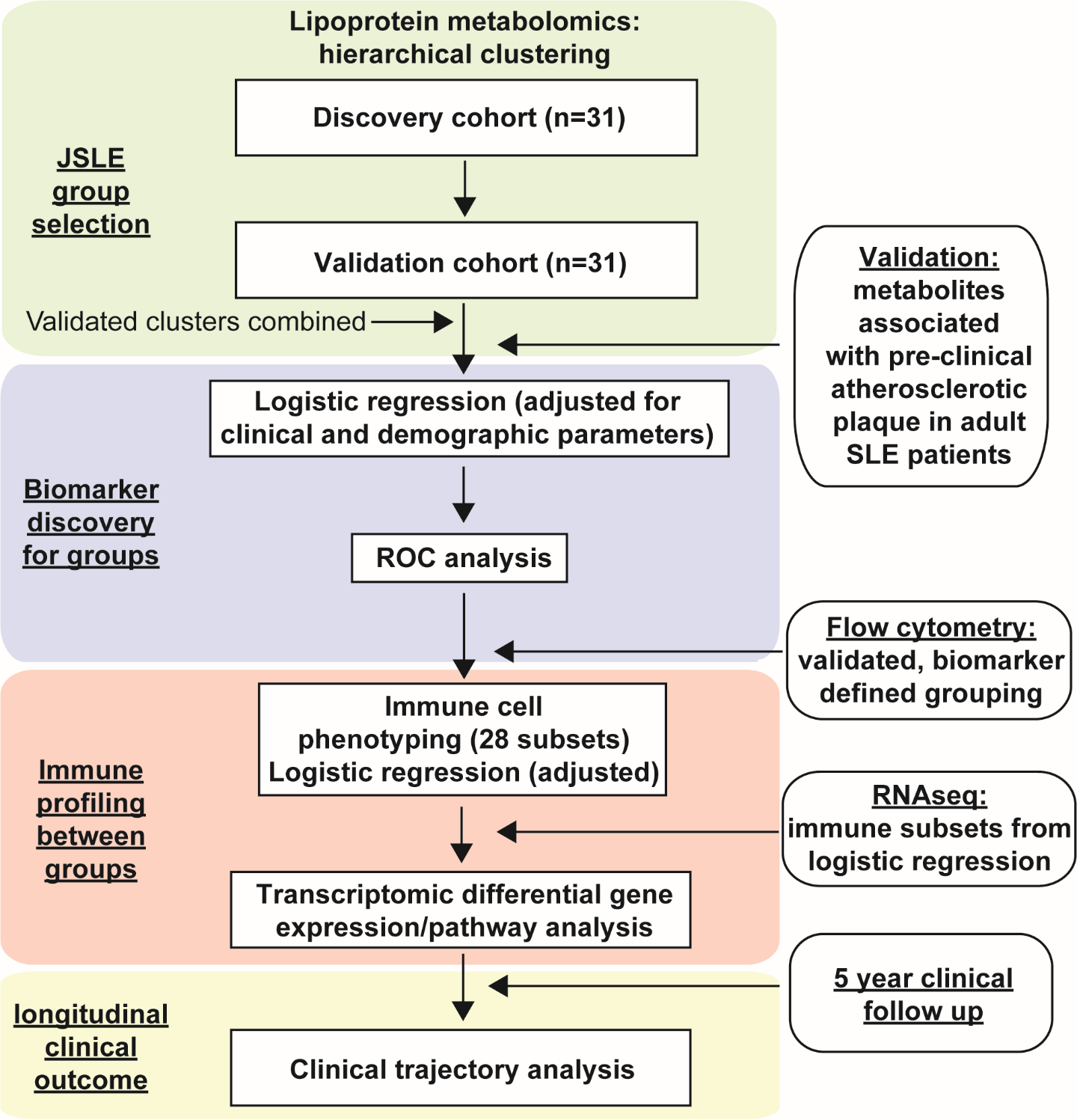
**

**Supplementary Figure 2. Study design and analysis plan flow diagram.** ROC, receiver operating characteristic; RNAseq, RNA sequencing.

**Supplementary Table 2. Metabolomics in Discovery JSLE cohort: Groups-1, -2 and -3**

| **Metabolite** | **Group 1, mean** | **Group 1, SD** | **Group 2, mean** | **Group 2, SD** | **Group 3, mean** | **Group 3, SD** |
| --- | --- | --- | --- | --- | --- | --- |
| Lipoproteins |  |  |  |  |  |  |
| XXL-VLDL-P (x10^-11^) | 14.5 | 18.0 | 1.04 | 2.24 | 0.454 | 1.11 |
| XL-VLDL-P (x10^-10^) | 8.83 | 11.8 | 0.549 | 1.22 | 0.208 | 0.510 |
| L-VLDL-P (x10^-9^) | 6.15 | 6.93 | 0.614 | 1.11 | 0.364 | 0.621 |
| M-VLDL-P (x10^-8^) | 2.22 | 1.77 | 0.613 | 0.303 | 0.6.11 | 0.217 |
| S-VLDL-P (x10^-8^) | 3.48 | 1.90 | 1.31 | 0.535 | 1.58 | 0.397 |
| XS-VLDL-P (x10^-8^) | 3.71 | 1.18 | 2.06 | 0.455 | 2.50 | 0.405 |
| IDL-P (x10^-8^) | 9.46 | 2.56 | 6.25 | 1.42 | 7.49 | 0.769 |
| L-LDL-P (x10^-7^) | 1.58 | 0.468 | 1.02 | 0.274 | 1.22 | 0.115 |
| M-LDL-P (x10^-8^) | 13.0 | 4.24 | 8.06 | 2.46 | 9.28 | 0.808 |
| S-LDL-P (x10^-8^) | 14.3 | 6.11 | 9.79 | 2.66 | 10.0 | 0.741 |
| XL-HDL-P (x10^-7^) | 2.04 | 1.63 | 4.83 | 1.34 | 1.82 | 1.09 |
| L-HDL-P (x10^-7^) | 5.63 | 3.14 | 14.3 | 2.41 | 10.3 | 3.02 |
| M-HDL-P (x10^-7^) | 12.9 | 6.55 | 19.5 | 2.47 | 18.4 | 3.43 |
| S-HDL-P (x10^-7^) | 44.3 | 5.34 | 45.6 | 3.71 | 46.2 | 5.27 |
| Lipoprotein diameters |  |  |  |  |  |  |
| VLDL-D | 37.2 | 1.54 | 35.5 | 0.979 | 35.0 | 0.628 |
| LDL-D | 23.6 | 0.163 | 23.6 | 0.104 | 23.8 | 0.08.17 |
| HDL-D | 9.62 | 0.235 | 10.1 | 0.152 | 9.83 | 0.131 |
| General cholesterol |  |  |  |  |  |  |
| Serum-C | 3.68 | 0.870 | 3.04 | 0.486 | 3.09 | 0.297 |
| VLDL-C | 0.728 | 0.369 | 0.256 | 0.0828 | 0.355 | 0.0791 |
| Remnant-C | 1.30 | 0.424 | 0.635 | 0.183 | 0.826 | 0.120 |
| LDL-C | 1.44 | 0.525 | 0.887 | 0.324 | 1.07 | 0.103 |
| HDL-C | 0.929 | 0.307 | 1.52 | 0.133 | 1.20 | 0.176 |
| HDL2-C | 0.486 | 0.255 | 1.04 | 0.126 | 0.745 | 0.161 |
| HDL3-C | 0.444 | 0.0877 | 0.472 | 0.0129 | 0.451 | 0.0219 |
| Esterified-C | 2.55 | 0.632 | 2.14 | 0.358 | 2.18 | 0.221 |
| Free-C | 1.12 | 0.259 | 0.901 | 0.130 | 0.907 | 0.0788 |
| Total-C | 1.84 | 0.318 | 1.74 | 0.185 | 1.61 | 0.211 |
| Glycerides and phospholipids |  |  |  |  |  |  |
| Serum-TG | 1.54 | 1.09 | 0.597 | 0.1.98 | 0.579 | 0.147 |
| VLDL-TG | 1.11 | 0.927 | 0.326 | 0.162 | 0.289 | 0.106 |
| LDL-TG | 0.167 | 0.0726 | 0.103 | 0.0185 | 0.105 | 0.0255 |
| HDL-TG | 0.149 | 0.0582 | 0.101 | 0.0166 | 0.114 | 0.0239 |
| TotPG | 1.50 | 0.274 | 1.42 | 0.167 | 1.30 | 0.222 |
| PC | 1.53 | 0.265 | 1.44 | 0.163 | 1.35 | 0.229 |
| SM | 0.357 | 0.0854 | 0.335 | 0.0382 | 0.321 | 0.0275 |
| Apolipoproteins |  |  |  |  |  |  |
| ApoA1 | 1.22 | 0.147 | 1.45 | 0.0844 | 1.31 | 0.134 |
| ApoB | 0.866 | 0.230 | 0.525 | 0.0984 | 0.586 | 0.0521 |
| ApoB/ApoA1 | 0.727 | 0.247 | 0.362 | 0.0651 | 0.451 | 0.0544 |
| Fatty Acids |  |  |  |  |  |  |
| UnSat | 1.17 | 0.0946 | 1.23 | 0.0394 | 1.22 | 0.0479 |
| DHA | 0.101 | 0.0290 | 0.0873 | 0.0170 | 0.0903 | 0.0205 |
| LA | 2.70 | 0.614 | 2.25 | 0.368 | 2.04 | 0.188 |
| FAw3 | 0.336 | 0.0786 | 0.259 | 0.0579 | 0.259 | 0.0554 |
| FAw6 | 3.20 | 0.632 | 2.71 | 0.374 | 2.54 | 0.197 |
| PUFA | 3.54 | 0.669 | 2.97 | 0.423 | 2.80 | 0.247 |
| MUFA | 3.08 | 1.46 | 1.86 | 0.383 | 1.71 | 0.286 |
| SFA | 3.59 | 1.10 | 2.65 | 0.329 | 2.62 | 0.376 |

**Supplementary Table 2. Metabolomics in Discovery JSLE cohort: Groups-1, -2 and -3.** Table displaying the mean (mmol/L or *g/L) and standard deviation for metabolites in discovery cohort Group-1, -2 and -3. Significant values between groups are shown in red. One-way ANOVA. P values are corrected for multiple testing (Holm-Sidak approach). See Figure 1a and b.

**Supplementary Figure 3. Patients in Gp1/G1A and Gp2/2A cluster together in a PCA analysis**

**
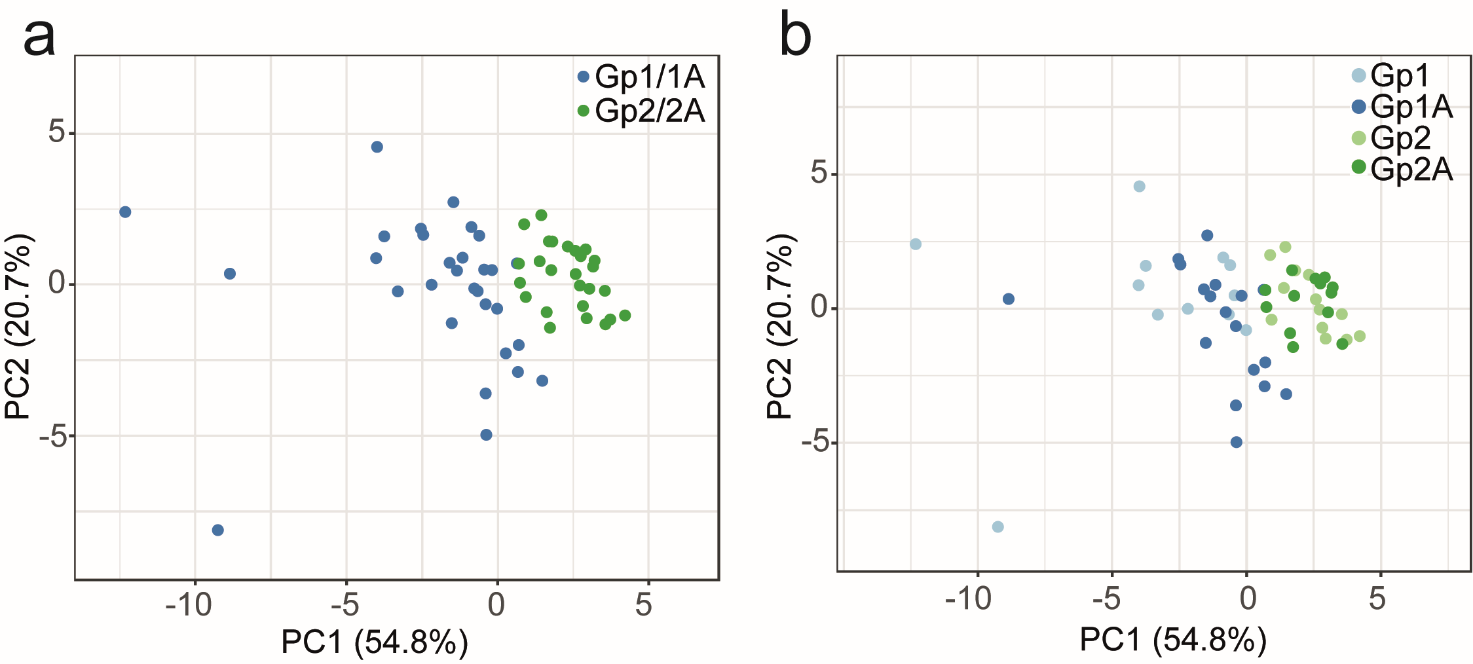
**

**Supplementary Figure 3. Patients in Gp1/G1A and Gp2/2A cluster together in a PCA analysis.** Principle component analysis (PCA) using the 19 lipoprotein clustering metabolites (Figure 1a and c) from **a)** Group-1/1A (n=30, blue) and Group-2/2A (n=26, green) and **b)** Discovery cohort Group-1 (n=12, light blue) and 2 (n=13, light green) and validation cohort Group-1A (n=18, dark blue) and 2A (n=13, dark green).

**Supplementary Table 3. Demographic and treatment information in the stratified JSLE patient groups (Discovery cohort).**

|  | **Group-1** | **Group-2** | **Group-3** |
| --- | --- | --- | --- |
| **Demographic** |  |  |  |
| Total number | 12 | 13 | 6 |
| Female:Male | 6:6 | 11:2 | 4:2 |
| Age, mean (range, SD) | 19.6 (14-23, 3) | 18.5 (15-23, 2.57) | 20 (17-25, 3.58) |
| BMI | 22.8 (20.6- 31.7) | 20.8 (19.2- 21.8) | 27.5 (22.3-32.2) |
| **Ethnicity, number (%):** |  |  |  |
| White | 4 (33) | 6 (46) | 1 (17) |
| Black | 3 (25) | 1 (8) | 3 (50) |
| Asian | 5 (42) | 5 (38) | 1 (17) |
| Other | 0 (0) | 1 (8) | 1 (17) |
| **Disease characteristics** |  |  |  |
| Age of diagnosis, mean (range, SD) | 11.7 (0-18, 5.47) | 11.7 (7-18, 3.07) | 11.7 (5-15, 3.88) |
| Disease duration, mean (range, SD)  (years) | 7.9 (0-21, 6.23) | 6.8 (2-13, 3.27) | 8.3 (3-14, 4.18) |
| SLEDAI, median (IQR) | 4 (2-7.5)  ) | 2 (1-5) | 1 (0-2.5) |
| SLEDAI >4, number (%) | 4 (33%) | 3 (23%) | 0 (0%) |
| **Current organ involvement, n (%):** |  |  |  |
| Neurological | 1 (8%) | 1 (8%) | 0 (0%) |
| Serositis | 1 (8%) | 3 (23%) | 3 (50%) |
| Cutaneous | 11 (92%) | 13 (100%) | 6 (100%) |
| Haematological | 5 (42%) | 8 (62%) | 0 (0%) |
| Musculoskeletal | 10 (83%) | 11 (85%) | 5 (83%) |
| Renal | 6 (50%) | 3 (23%) | 0 (0%) |
| **Serology [median (IQR)]:** |  |  |  |
| dsDNA (IU/mL) (NR=<50) | 162 (20-390) | 60 (2.5-228) | 6 (1.5-99) |
| Positive ENA (number, %) | 5 (42%) | 8 (62%) | 5 (83%) |
| Anti-CL IgM (MPL) (NR=0-10) | 2.9 (1.2-3.1) | 3.8 (1.4-6.1) | 2.2 (0.8-4.9) |
| Anti_CL IgG (GPL) (NR=0-20) | 2.4 (1.4-8.5) | 1.1 (0.4-2.5) | 1.5 (1-5.4) |
| hsCRP (mg/L) (NR<5) | 1.3 (0.6-4.38) | 0.6 (0.6-1.6) | 0.9 (0.6-11.4) |
| C3 (g/L) (NR=0.9-1.8) (mean (range, SD) | 0.85 (0.33-1.44, 0.38) | 0.95 (0.58-1.34, 0.23) | 1.28 (1.02-1.64, 0.22) |
| LC (10^9^/L) (NR=1.3-3.5) | 1.69 (0.59-3.8) | 1.5 (0.85-3.08) | 1.36 (1.14-1.8) |
| ESR (mm/hr) (NR=<20) | 30 (2-127) | 5 (2-44) | 22 (2-78) |
| Urine protein:creatinine (mg/mmol)(NR=0-13) | 7 (4-15) | 7 (6-12) | 11.5 (9.25-21) |
| **Clinical lipids [median (IQR)]:** |  |  |  |
| Cholesterol (NR<5mmol/L) | 4.2 (3.8-4.6) | 3.8 (3.4-4.2) | 3.8 (3.5-4.2) |
| Triglycerides (NR<3mmol/L) | 1 (0.8-2.1) | 0.8 (0.5-1.2) | 0.6 (0.5-0.8) |
| HDL-C (NR>1mmol/L) | 1.1 (0.9-1.3) | 1.6 (1.4-1.7) | 1.7 (1.4-1.9) |
| LDL-C (NR<3mmol/L) | 2.2 (2-3.1) | 1.7 (1.5-2.1) | 1.9 (1.7-2.1) |
| Cholesterol:HDL (NR<4) | 4 (2.5-4.7) | 2.5 (2.2-2.7) | 2.3 (2.2-2.5) |
| Non-HDL-C (NR<4mmol/L) (Nightingale) | 3.1 (2.5-3.6) | 2.2 (1.8-2.6) | 2.3 (2.2-2.6) |
| **Current treatment (%)** |  |  |  |
| Prednisolone | 50 | 46 | 50 |
| Hydroxychloroquine | 83 | 92 | 83 |
| Methotrexate | 8 | 8 | 33 |
| Mycophenolate | 33 | 69 | 50 |
| Azathioprine | 25 | 8 | 33 |
| Vitamin D | 8 | 23 | 50 |
| **Past treatment (%)** |  |  |  |
| Rituximab in the last year | 0 | 0 | 0 |
| Rituximab ever | 3 | 0 | 0 |
| Cyclophosphamide in the last year | 0 | 0 | 0 |

**Supplementary Table 3. Demographic and treatment information in the stratified JSLE patient groups.** Demographic and treatment information from 31 JSLE patients stratified into 3 groups by metabolomics analysis. For patients the SLE Disease Activity Index (SLEDAI) was calculated, a score greater than 4 represents active disease. Other common clinical measures of disease are shown as well as treatments. Normal ranges for lipid measures are relevant for healthy adults. Of note, only two patients (6.4%) had clinically high LDL cholesterol levels, three patients (9.6%) clinically low HDL cholesterol levels, three patients (9.6%) had elevated TG levels and two patients (6.4%) had elevated non-HDL cholesterol levels.

Abbreviations: NR: Normal ranges, BMI: Body mass index, SLEDAI: Systemic Lupus Erythematosus Disease Activity Index, ENA: Extractable nuclear antigens, Anti-CL: Anti-cardiolipin, dsDNA: Anti-double-stranded-DNA antibodies, hsCRP: high sensitivity C-reactive protein, C3: Complement component 3, LC: Lymphocyte count, ESR, erythrocyte sedimentation rate, HDL-C: High density lipoprotein cholesterol, LDL-C: Low density lipoprotein cholesterol.

**Supplementary Table 4. Demographic and treatment information in the two JSLE validation cohort groups**.

|  | **Group-1A** | **Group-2A** |
| --- | --- | --- |
| **Demographic** |  |  |
| Total number | 18 | 13 |
| Female:Male | 18:0 | 12:1 |
| Age, mean (range, SD) | 19.3 (13-24, 2.86) | 19.4 (16-22, 2.33) |
| BMI | 24.3 (21.7- 30.1) | 21.2 (18.5- 23.4) |
| **Ethnicity, number (%):** |  |  |
| White | 5 (28) | 3 (23) |
| Black | 6 (33) | 5 (38) |
| Asian | 6 (33) | 3 (23) |
| Other | 1 (6) | 2 (15) |
| **Disease characteristics** |  |  |
| Age of diagnosis, mean (range, SD) | 12 (8-18, 3.66) | 13.3 (8-17, 2.67) |
| Disease duration, mean (range, SD)  (years) | 7.2 (0-14, 3.99) | 6 (0-11, 3.27) |
| SLEDAI, median (IQR) | 2 (0-3.3) | 0 (0-2) |
| SLEDAI >4, number (%) | 1 (6%) | 0 (0%) |
| **Current organ involvement, n (%):** |  |  |
| Neurological | 6 (33%) | 3 (23%) |
| Serositis | 0 (0%) | 0 (0%) |
| Cutaneous | 14 (78%) | 11 (85%) |
| Haematological | 5 (28%) | 7 (54%) |
| Musculoskeletal | 15 (83%) | 9 (69%) |
| Renal | 7 (39%) | 2 (15%) |
| **Serology [median (IQR)]:** |  |  |
| dsDNA (IU/mL) (NR=<50) | 36 (14-67.5) | 5 (0-29.5) |
| Positive ENA (number, %) | 7 (39%) | 11 (85%) |
| Anti-CL IgM (MPL) (NR=0-10) | 2.1 (1.3-2.5) | 2.1 (1.5-7) |
| Anti_CL IgG (GPL) (NR=0-20) | 1.25 (0.5-3) | 1.3 (0.4-2.1) |
| hsCRP (mg/L) (NR<5) | 0.9 (0.6-4.65) | 0.6 (0.6-1.10) |
| C3 (g/L) (NR=0.9-1.8) (mean (range, SD)) | 0.98 (0.63-1.47, 0.26) | 0.99 (0.57-1.28, 0.23) |
| LC (10^9^/L) (NR=1.3-3.5) | 1.19 (0.8-3.6) | 1.12 (1-1.4) |
| ESR (mm/hr) (NR=<20) | 24 (2-34.5) | 7 (2-27) |
| Urine protein:creatinine ratio (mg/mmol) (NR=0-13) | 9 (6-12.75) | 7 (5-8) |
| **Clinical lipids [median (IQR)]:** |  |  |
| Cholesterol (NR<5mmol/L) | 3.8 (3.2-4.9) | 4 (3.2-4.7) |
| Triglycerides (NR<3mmol/L) | 0.9 (0.8-1.4) | 0.5 (0.4-0.8) |
| HDL-C (NR>1mmol/L) | 1.2 (1.1-1.5) | 1.8 (1.4-2) |
| LDL-C (NR<3mmol/L) | 2.4 (1.7-2.8) | 1.9 (1.3-2.3) |
| Cholesterol:HDL (NR<4) | 3.1 (2.9-3.6) | 2.2 (1.8-2.8) |
| Non-HDL-C (NR<4mmol/L) (Nightingale) | 2.7 (1.9-3.3) | 2.3 (2.0-2.5) |
| **Current treatment (%)** |  |  |
| Prednisolone | 22 | 54 |
| Hydroxychloroquine | 89 | 100 |
| Methotrexate | 22 | 0 |
| Mycophenolate | 22 | 38 |
| Azathioprine | 17 | 38 |
| Vitamin D | 11 | 31 |
| **Past treatment (%)** |  |  |
| Rituximab in the last year | 0 | 0 |
| Rituximab ever | 1 | 0 |
| Cyclophosphamide in the last year | 0 | 0 |

**Supplementary Table 4. Demographic and treatment information between the 2 JSLE validation cohort groups**. Demographic and treatment information displayed from 31 JSLE patients (Validation cohort) across 2 validated groups stratified by metabolomics (discovery cohort Group-3 was not validated). SLEDAI score was calculated, a score greater than 4 represents active disease. Other common clinical measures of disease are shown as well as treatments. Normal ranges for lipid measures are relevant for healthy adults.

Abbreviations: NR: Normal ranges, BMI: Body mass index, SLEDAI: Systemic Lupus Erythematosus Disease Activity Index, ENA: Extractable nuclear antigens, Anti-CL: Anti-cardiolipin, dsDNA: Anti-double-stranded-DNA antibodies, hsCRP: high sensitivity C-reactive protein, C3: Complement component 3, LC: Lymphocyte count, ESR, erythrocyte sedimentation rate, HDL-C: High density lipoprotein cholesterol, LDL-C: Low density lipoprotein cholesterol.

**Supplementary Figure 4. Odds ratios of non-lipid metabolites between high and low ApoB:ApoA1 ratio groups adjusting for clinical parameters**

**
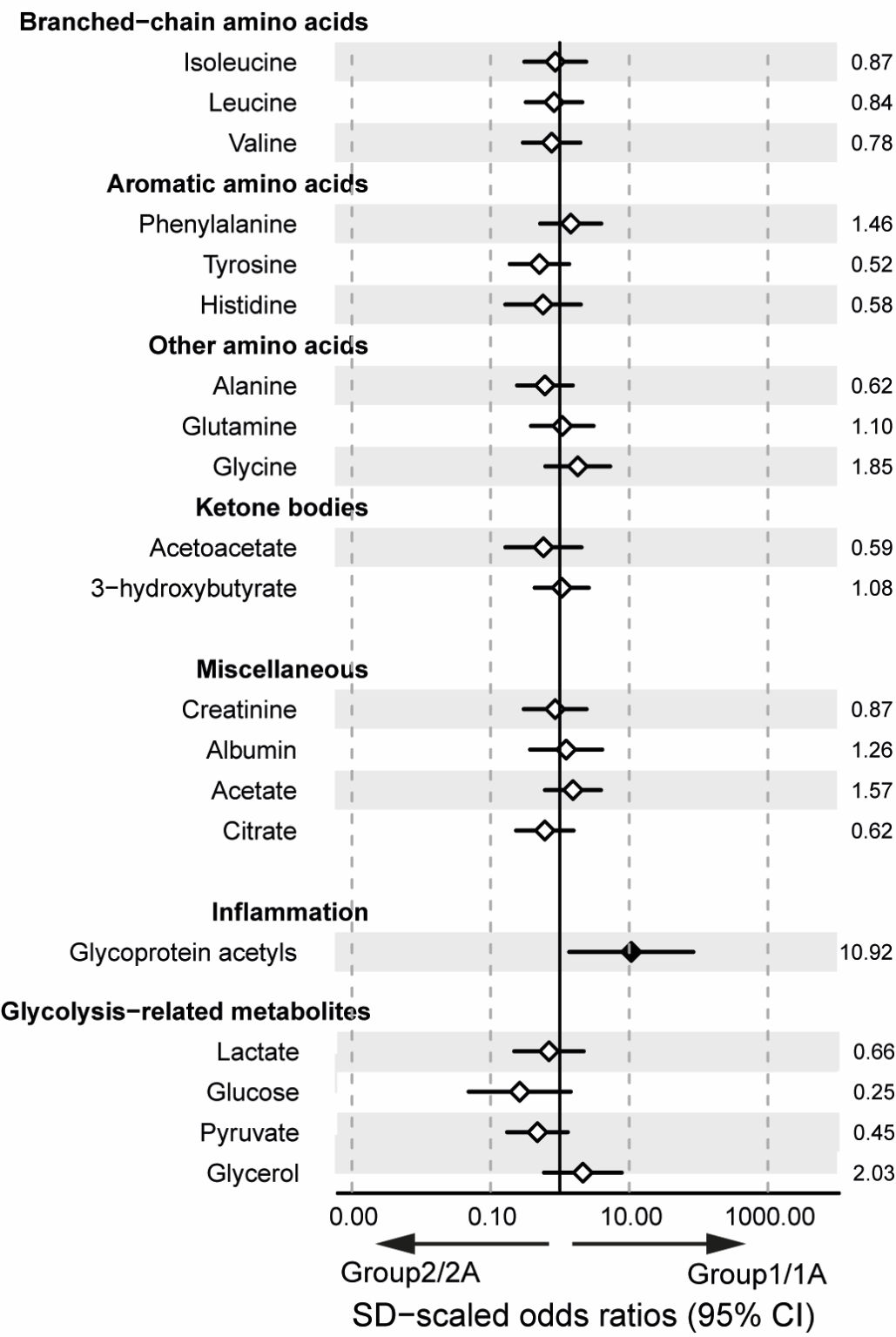
**

**Supplementary Figure 4. Odds ratios of non-lipid metabolites between Group-1/1A and Group-2/2A adjusting for clinical parameters.** Forest plot displaying the odds ratios of blood non-lipid metabolites between Group-1/1A (high ApoB:ApoA1 ratio) and Group-2/2A (low ApoB:ApoA1 ratio) adjusted for age, sex, treatments, BMI and disease parameters. Significances between groups are displayed as a filled in point. Metabolites significantly altered by clinical and demographic parameters are shown in Supplemental Data Table 1.

**Supplementary Table 5. Significant metabolites distinguishing high and low ApoB:ApoA1 ratio groups following adjustment for all clinical data, treatment and BMI**

| **Metabolite** | **P value** | **Adjusted P Value** |
| --- | --- | --- |
| L-HDL-CE | 0.0000741 | 0.016753499 |
| L-HDL-C | 0.0000745 | 0.016769923 |
| L-HDL-FC | 0.0000789 | 0.017674052 |
| L-HDL-L | 0.0000799 | 0.017817574 |
| L-HDL-P | 0.000083 | 0.018420992 |
| L-HDL-PL | 0.0000832 | 0.018420992 |
| S-VLDL-CE | 0.000109787 | 0.024079404 |
| ApoB/ApoA1 | 0.000122933 | 0.026804083 |
| HDL2-C | 0.000129007 | 0.027984347 |
| HDL-C | 0.00014798 | 0.031890442 |
| HDL-D | 0.000168289 | 0.036025165 |

**Supplementary Table 5. Significant metabolites distinguishing between high and low ApoB:ApoA1 ratio groups following adjustment for all clinical data, treatment and BMI.** Significant metabolites between Group-1/1A and Group-2/2A following normalisation for clinical parameters, demographic information and BMI (Corrected for multiple testing using Holm-Sidak). See Figure 2 and Figure 3a.

**Supplementary Table 6. Principle component analysis factor loadings of the significant metabolites distinguishing high and low ApoB:ApoA1 ratio groups**

| **Metabolite** | **PC1 (84.7%)** | **PC2 (9.2%)** |
| --- | --- | --- |
| **L-HDL-L** | -0.33 | 0 |
| **L-HDL-CE** | -0.32 | 0.04 |
| **L-HDL-C** | -0.32 | 0.04 |
| **L-HDL-FC** | -0.32 | 0.05 |
| **L-HDL-PL** | -0.32 | -0.02 |
| **L-HDL-P** | -0.32 | -0.01 |
| **HDL-C** | -0.32 | -0.15 |
| **HDL-D** | -0.31 | -0.05 |
| **HDL2-C** | -0.13 | -0.89 |
| **S-VLDL-CE** | 0.27 | -0.41 |
| **ApoB/ApoA1** | 0.29 | -0.13 |

**Supplementary Table 6. Principle component analysis factor loadings of the significant metabolites distinguishing high and low ApoB:ApoA1 ratio groups.** Patients were clustered by principle component (PCA) analysis using metabolites that were significantly different between Group-1/1A and Group-2/2A (following normalisation for clinical parameters and adjustment for multiple testing). Table displays the PCA factor loading values for each metabolite in principle component (PC) 1 and 2; these PC’s explain 84.7% and 9.2% of the total patient variance, respectively. See Figure 3b.

**Supplementary Figure 5. ROC curve analysis of top differentially expressed metabolites between Group-1/1A and Group-2/2A**

**
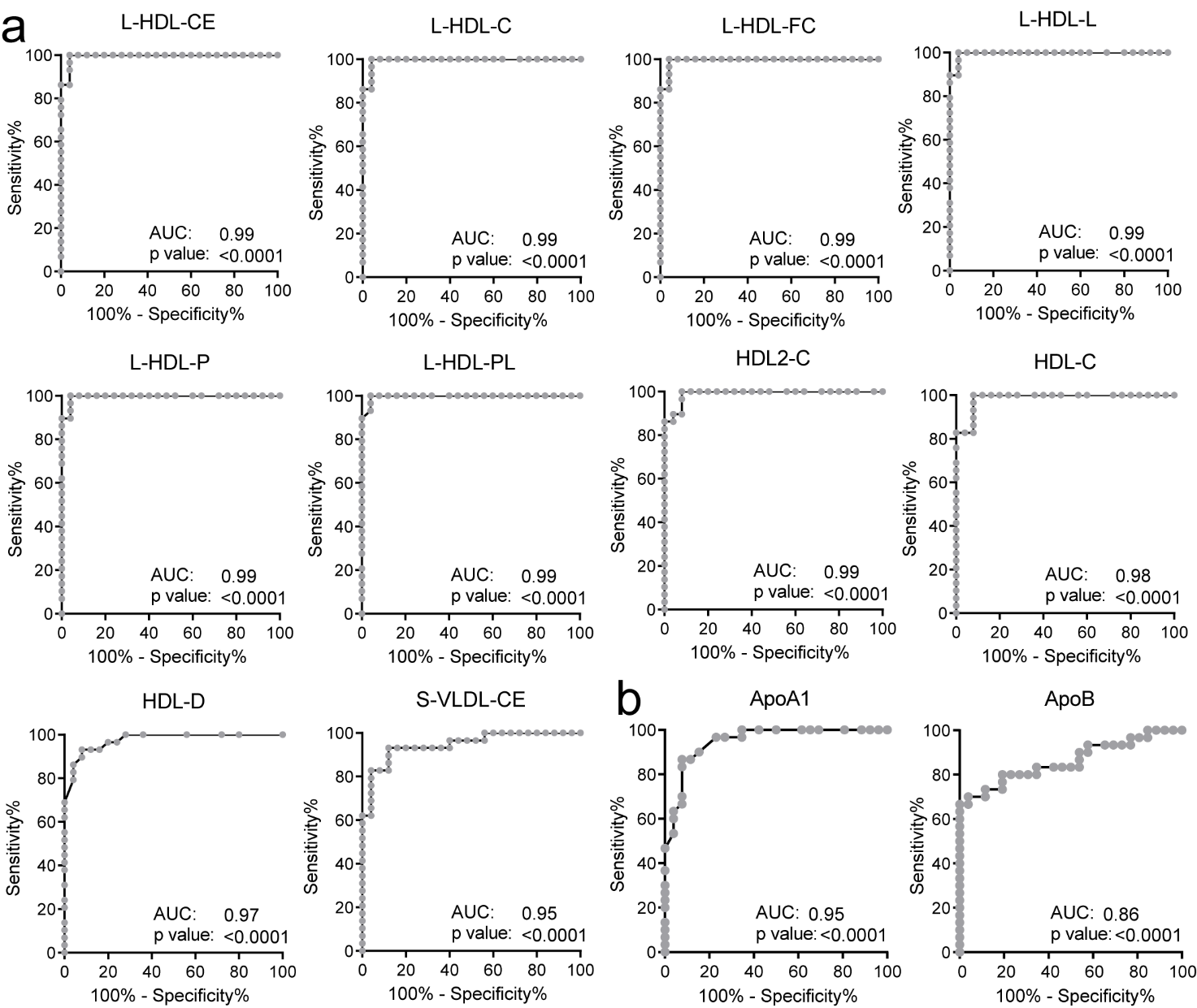
**

**Supplementary Figure 5. ROC analysis of top differentially expressed metabolites between Group-1/1A and Group-2/2A.** ROC curve analysis of **(a)** top differentially expressed metabolites (see Fig. 3a-c and Supplemental Table 5) and **(b)** ApoA1 and ApoB. Area under the curve (AUC) and p value shown. See Supplementary Data Table S1.

**
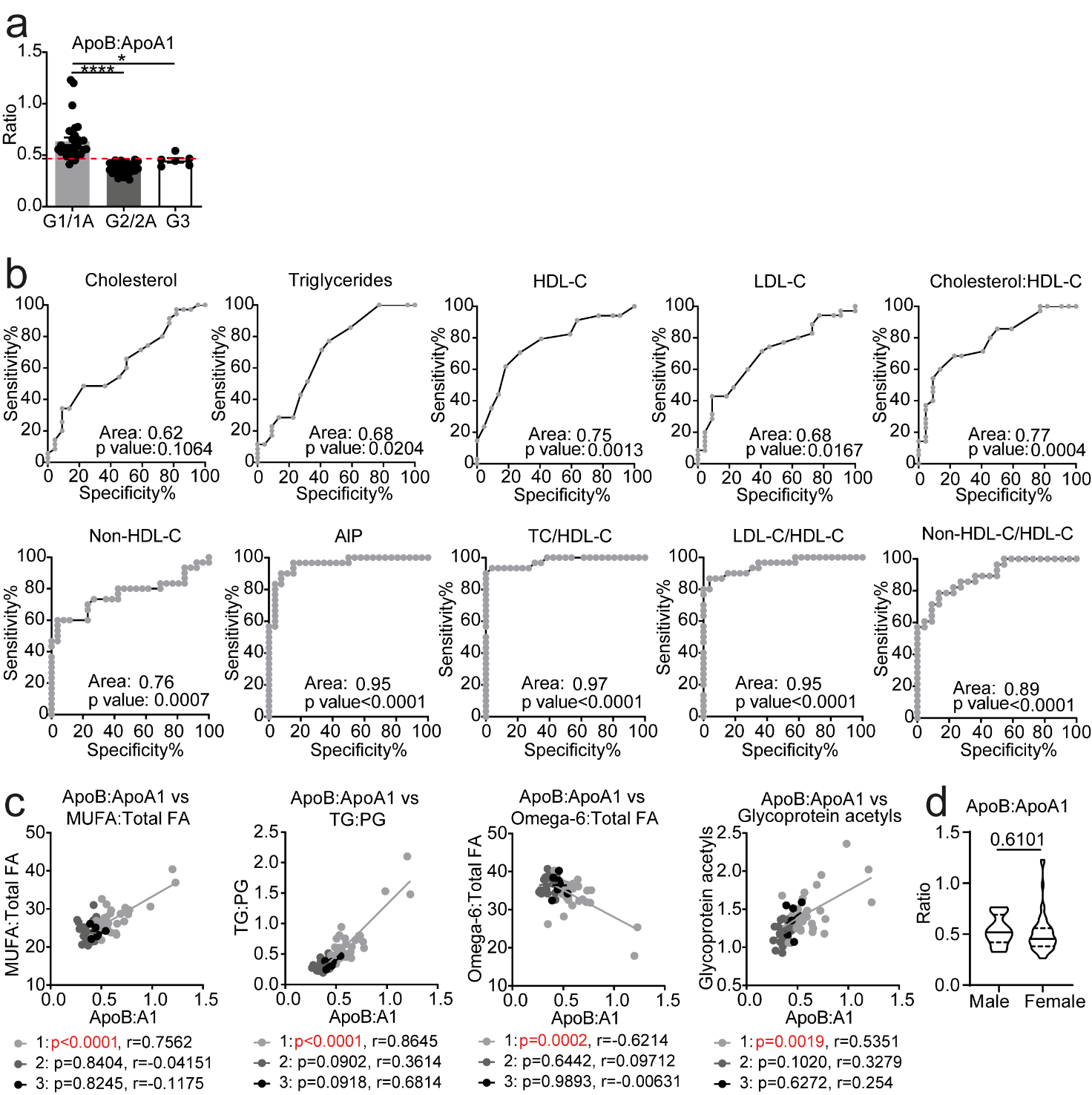
Supplementary Figure 6. ApoB:ApoA1 ratio correlates with biomarkers associated with pre-clinical plaque in Group-1/1A but not Group-2/2A or 3**

**Supplementary Figure 6. ApoB:ApoA1 ratio correlates with biomarkers associated with pre-clinical plaque in Group-1/1A but not Group-2/2A or 3. (a)** Assessment of ApoB:ApoA1 ratio across three patient groups: discovery and validation JSLE cohort data were combined (Group-1/1A (n=30), Group-2/2A (n=26)) and discovery cohort Group 3 (n=6). Red line represents the mean ApoB:ApoA1 ratio of age and sex matched healthy controls (n=32; 15 males, 17 females). **(b)** ROC curve analysis of lipid and atherogenic index measures available from standard clinical laboratory measurements between Group-1/1A and Group-2/2A. Area under curve (AUC) and p value are displayed. See Supplementary Tables 3 and 4. **(c)** Correlations between serum ApoB:ApoA1 levels and biomarkers associated with pre-clinical atherosclerotic plaque in adult SLE patients(26). Pearson’s correlation coefficient (r) and significance were determined using a 95% confidence interval. Statistically significant p values are displayed in red. AIP: Atherogenic index of plasma, FA: Fatty acids, HDL: high density lipoprotein, LDL: low density lipoprotein, MUFA: Monounsaturated fatty acids, TC: total cholesterol, TG: Triglycerides, PG: Phosphoglycerides. **(d)** Violin plot displaying ApoB:ApoA1 ratio measures between JSLE males (n=11) and females (n=51).

**Supplementary Figure 7. ROC curve analysis of significantly altered immune cell subsets between Group-1/1A and Group-2/2A**

**
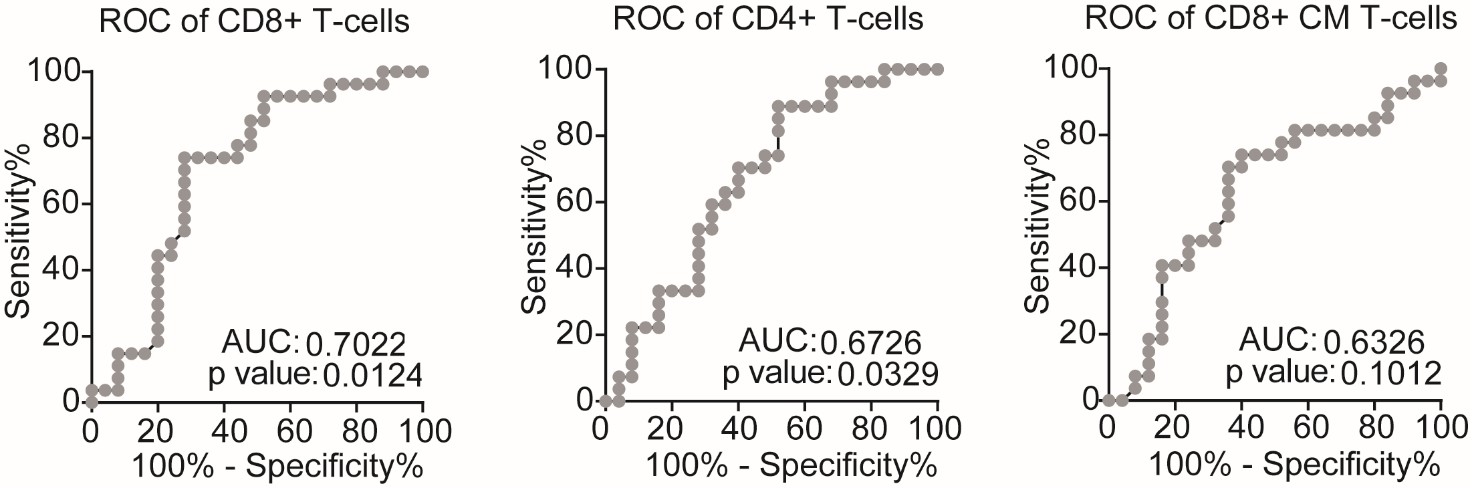
**

**Supplementary Figure 7. ROC curve analysis of significantly altered immune cell subsets between Group-1/1A and Group-2/2A.** ROC curve analysis of significantly altered immune cell subsets (see Fig. 4a-b) between JSLE patients in Group-1/1A vs Group-2/2A. Area under curve (AUC) and p value is displayed. Respective sensitivity/specificity for CD8+ T-cells, CD4+ T-cells and CD8+ CM T-cells are 74.07/ 72.00, 70.37/ 60.00 and 70.37/ 64.00. CM=central memory. See Supplementary Data Table S2.

**Supplementary Table 7.** List of DEGs in CD4+ and CD8+ T-cells from JSLE patients with High vs Low ApoB:ApoA1 ratios.

| **CD4** | **CD8** | | | | | |
| --- | --- | --- | --- | --- | --- | --- |
| DDX3Y | MTRNR2L1 | PTPN7 | DDX39A | MAPK8 | ERFL | IRS2 |
| MTRNR2L1 | CD38 | DTX3L | GAPDH | TBL1X | ACACB | SLC22A17 |
| NR4A1 | PRKY | PARP14 | WDR76 | BEX4 | ASPH | SSBP2 |
| PRKY | HLA-DQB1 | HSH2D | ACTG1P4 | ITGB2-AS1 | MFGE8 | ROBO1 |
| SOX4 | RSAD2 | DDX60L | PHF11 | ESYT2 | ITGA6 | APP |
| HCG4B | GFOD1 | GEN1 | PPP5C | ABCD2 | GABBR1 | CDH23 |
| HPGD | CCR5 | MARK4 | DNAJB11 | GRAMD1A | HIPK2 | FCGBP |
| RHOB | CMPK2 | ANXA2P2 | HSP90AA1 | SCPEP1 | DUSP16 |  |
| FSBP | IFI44 | APOBEC3F | IFITM1 | CBX7 | FAM216A |  |
| PTPRM | ISG15 | NFKBID | PFN1 | CAMK4 | DPEP2 |  |
| ZNF117 | LGALS9 | LMNB1 | FERMT3 | ERC1 | BACH2 |  |
| MIR6891 | TYMP | TIMELESS | YARS | PWAR5 | SETD1B |  |
| ADAM23 | HLA-DRA | ATP1A3 | COMMD4 | STAG3L1 | CEP68 |  |
| DUSP10 | OASL | CD74 | RNF213 | TRIM66 | ZNF395 |  |
| JUN | KLRA1P | ADA | HSPB11 | CUX1 | C19orf71 |  |
| PPIF | MX1 | ATP1B3 | DCLRE1C | RPL5 | ZBTB18 |  |
| ARRDC4 | EPSTI1 | DNAJA1 | ACTB | EIF3E | LEF1 |  |
| ZNRF1 | USP18 | CASP3 | MTHFD1 | ZNF609 | TANC2 |  |
| SLC19A1 | PALM2-AKAP2 | NAPRT | GSR | FLJ32255 | TFAP4 |  |
| CAPN5 | CIITA | HELZ2 | GBA2 | LOC100996720 | LZTS3 |  |
| GPSM1 | CDCA7 | REC8 | ZNFX1 | EIF2D | KCNQ1 |  |
| MAPK11 | LAG3 | UBE2L6 | IPPK | HPCAL1 | LOC102723566 |  |
| ADCY4 | GZMA | PDPR | PSMC4 | ISYNA1 | HABP4 |  |
| MCM2 | IFI6 | CISD3 | MCM5 | AKT3 | LMTK3 |  |
| TPCN2 | EOMES | SP100 | PSMD8 | UXT | RALGPS2 |  |
| PSD | HLA-DRB1 | IGHMBP2 | FANCG | PLXNA1 | RNF130 |  |
| SLC26A1 | PARP9 | OPTN | TRAF2 | CPQ | PDCD4-AS1 |  |
| PUS7 | OAS3 | ITGAL | RPS6KA1 | WHAMMP2 | IL7R |  |
| NAPRT | HCG4B | FANCA | MRPL54 | THRA | MAML2 |  |
| ADA | LOC100130872 | STMN1 | COX5A | MLXIP | SVIL |  |
| CHAF1A | PLSCR1 | DPP3 | PSMA6 | CNST | TRABD2A |  |
| NPHP4 | HERC5 | EBP | HAUS1 | FOXP1 | KCNC4 |  |
| PEAK1 | MT2A | BCL2L1 | PSMB3 | TSTD1 | ZNF154 |  |
| ADCY3 | CMC1 | ZBP1 | TTLL3 | ZDBF2 | ZNF815P |  |
| PRR29 | SCD | STAT2 | STIMATE | SUPT3H | FER |  |
| SREBF2-AS1 | MCM4 | SH3BP2 | ELMO2 | SELENOM | LMO7 |  |
| CENPO | CTNNA1 | EIF4G3 | PSMD14 | CEP170 | MXI1 |  |
| PI4KAP2 | HNRNPLL | BISPR | PSMC3 | ABLIM1 | RAB43 |  |
| CEP131 | GRAMD4 | MT1F | NPIPB3 | GAS5 | AMIGO1 |  |
| TRAP1 | TP53INP1 | NMI | PML | ALKBH7 | SNHG5 |  |
| TET3 | ABI3 | PDXP | MRPL28 | RPS5 | IL24 |  |
| TMEM116 | DDX60 | NPHP4 | MAP4K1 | ZNF813 | ARHGAP5 |  |
| LRIG1 | TREX1 | PARP12 | GYS1 | ARRDC3 | OBSCN |  |
| ZNF329 | KLF6 | CYCS | SNF8 | PBX3 | USP53 |  |
| LINC00674 | FAS | CARD16 | PPP2R3C | ZNF550 | PKIA |  |
| TMEM71 | SLC1A5 | KIAA0930 | PSMA4 | ANKRD46 | KLF7 |  |
| ZBTB38 | TIGIT | GCH1 | ACSL5 | RPS23 | PLD6 |  |
| TXLNG | MAPK11 | APOL6 | SRPK1 | AKTIP | MIR600HG |  |
| RIC3 | EZH2 | RILPL2 | ADAR | RAB3IP | NELL2 |  |
| ZNF266 | MYO1C | ACOT9 | ARPC2 | LOC339192 | SESN3 |  |
| APP | MCOLN2 | CASP1 | MEA1 | RECK | ZIK1 |  |
| RESF1 | SUSD1 | MAPK7 | IRF9 | FAM102A | LINGO3 |  |
| RCBTB2 | KLF10 | TRIM25 | SNAP29 | PHF7 | ZNF204P |  |
| ACSL6 | RAP1GAP2 | EIF4E3 | SMC6 | RAB39B | PCSK5 |  |
| RALGPS1 | TRAF4 | MIS18BP1 | NPIPB12 | SVIP | AXIN2 |  |
| ZNF737 | LAP3 | NABP2 | RTF1 | ACSL6 | SLC9A3 |  |
| ZNF204P | CTSC | SLC38A5 | CCZ1B | MPP7 | IRS1 |  |
| ZNF568 | SMC4 | IFITM2 | RNF31 | SORL1 | SH3PXD2A |  |
| IL7R | EIF2AK2 | MYD88 | KIF1C | SATB1-AS1 | MEGF6 |  |
| CA5B | APOBEC3D | IKZF3 | POLE | DMXL2 | RCAN3 |  |
| ITGB2-AS1 | IFI16 | STAT1 | NOD1 | CCDC88A | CNKSR2 |  |
| NELL2 | B4GALT5 | SCLT1 | SRSF8 | EFEMP2 | VSIG1 |  |
| ZNF23 | IFI35 | PTOV1-AS1 | ZDHHC2 | SLC12A6 | ADAMTS10 |  |
| LAPTM4B | FBXO6 | POTEM | BBS2 | ACVR1B | MYO15B |  |
| OBI1 | PDE1B | TAF1B | SMPD3 | SAMD1 | NR3C2 |  |
| VSIG1 | ANXA2 | PSME2 | PABPC1 | MTERF4 | ROBO3 |  |
| ZSCAN18 | JPT1 | LINC00265 | ASF1A | SATB1 | ZNF844 |  |
| NACC2 | ARPC5L | GALM | HSDL1 | TGFBR2 | SNED1 |  |
| ACP6 | IFIH1 | SQOR | SKI | LINC02361 | VASH1 |  |
| ZFP82 | SH2D1A | BST2 | ST3GAL1 | TMEM38B | EML5 |  |
| SPEF2 | FANCI | MRPL14 | SLC25A37 | PDE7A | ZSCAN18 |  |
| PTCH1 | JAK2 | HEG1 | DGCR2 | AGRN | IGF1R |  |
| ZNF626 | CHAF1A | WSB2 | COQ8A | GSTM2 | PRKXP1 |  |
| MMP24-AS1-EDEM2 | YWHAH | TUBB | ST13 | TPM2 | ZNF818P |  |
| PLAG1 | SAMD3 | ADGRE5 | UFL1 | PDE3B | GCNT4 |  |
| PIGY | NFKBIB | SMS | PCED1B-AS1 | CTSF | INF2 |  |
| FCGBP | PPIF | USB1 | ITPKB | RETREG1 | PLAG1 |  |
| SLC40A1 | LY6E | PGAM4 | MPP6 | A1BG | SLC9A3-AS1 |  |
| ZNF208 | INTS7 | SLC6A6 | TMEM106B | ENGASE | KCNQ5 |  |
| CDC14B | CLIC1 | POMP | NAP1L1 | APBB1 | NBEA |  |
| PACSIN1 | ACTA1 | TDRD7 | TAB2 | SLC26A11 | SHANK1 |  |
| TMEM45B | SNORD140 | HYPK | PCYOX1 | S1PR1 | NSG1 |  |

**Supplementary Table 7. List of DEGs in CD4+ and CD8+ T-cells from JSLE patients with High vs Low ApoB:ApoA1 ratios:** List of genes significantly altered in expression between high and low ApoB:A1 ratio JSLE patients. Upregulation in high ApoB:ApoA1 ratio is displayed in green and downregulated in blue.


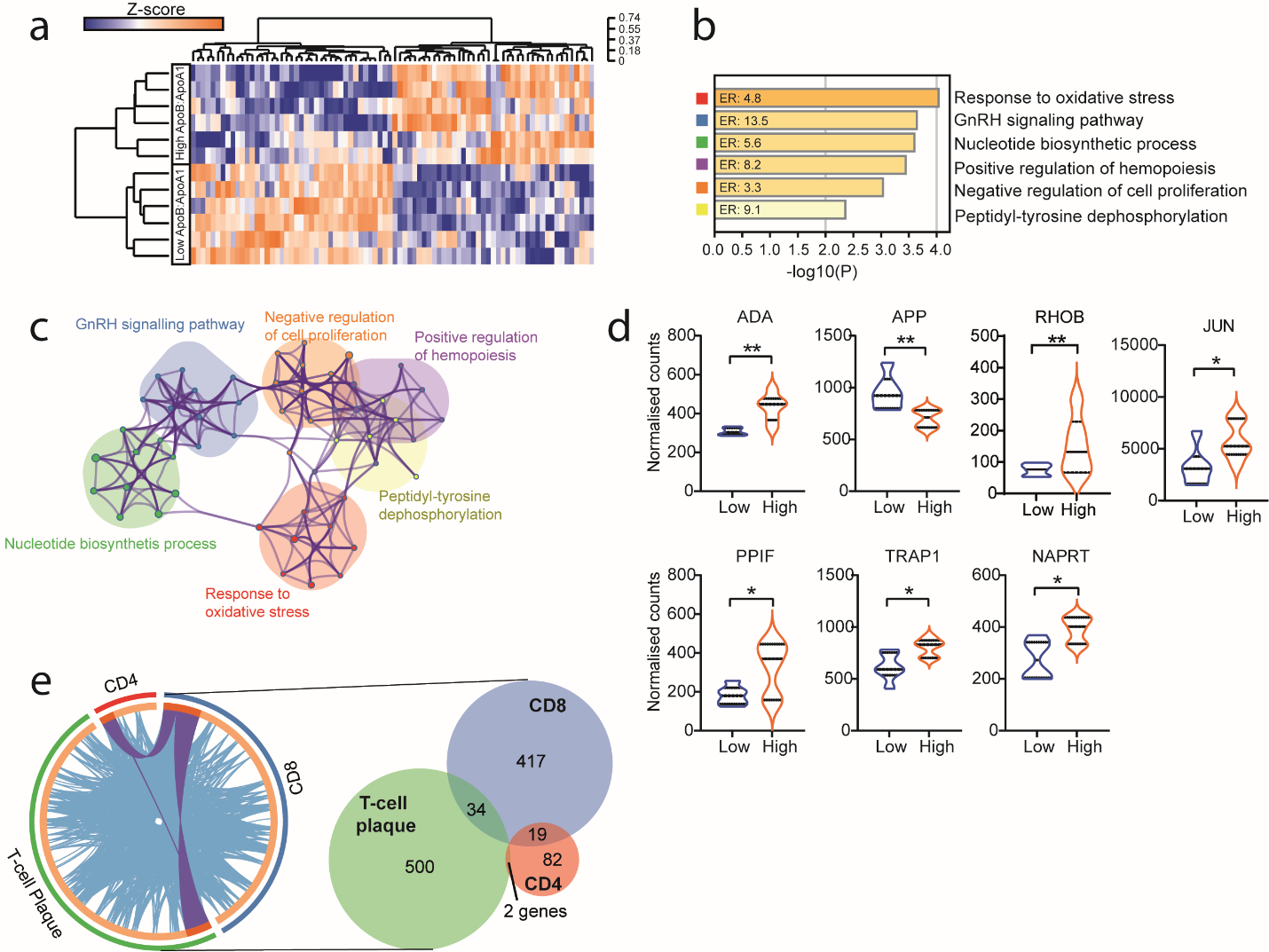
**Supplementary Figure 8: DEGs in T-cells from JSLE patients with High vs Low ApoB:ApoA1 ratios.**

**Supplementary Figure 8: DEGs in T-cells from JSLE patients with High vs Low ApoB:ApoA1 ratios.**

FACS-sorted CD4+ T-cells (n=6/group) from JSLE patients with high and low ApoB:ApoA1 ratio were analysed by RNA-sequencing and whole genome expression compared between the groups (See Figure 4d). **(a)** Clustered heatmap of normalised gene counts of significantly altered genes with adjusted p-value threshold (<0.01). **(b)** Bar charts plotting cluster significance and enrichment ratio (ER) of enriched pathway ontology terms between high and low ApoB:ApoA1 JSLE patients. **(c)** Network diagram illustrating significantly enriched genetic pathway ontology terms between high and low ApoB:ApoA1 JSLE patients. Similar terms with a high degree of redundancy were clustered into groups as depicted. Each node represents a significantly enriched term, with node size proportional to the number of input genes annotated with this term. **(d)** Violin plots comparing normalised gene counts of genes associated with response to oxidative stress (see b). Unpaired t test, *P<0.05, **=P<0.01. **(e)** Circos plot (Metascape) and Venn diagram showing the proportion of genes also regulated in previously published T-cell plaque datasets(34). The Circos plot shows how genes from the input gene lists overlap. On the outside, each arc colour represents the identity of each gene list. On the inside, dark orange color represents the genes that appear in multiple lists and light orange color represents genes that are unique to that gene list. Purple lines link the same gene that are shared by multiple gene lists. Blue lines link the different genes where they fall into the same ontology term (the term has to be statistically significantly enriched and with size no larger than 100).

**Supplementary Table 8. List of overlapping DEGs between T-cells from JSLE patients with High vs Low ApoB:ApoA1 ratios and human atherosclerotic plaque.**

| **Gene ID** | **JSLE (ApoB:ApoA1)** | | **Human atherosclerotic plaque** | **Description** |
| --- | --- | --- | --- | --- |
|  | **CD4** | **CD8** | **T-cell** |  |
| HCG4B |  |  |  | HLA complex group 4B |
| PRKY |  |  |  | protein kinase Y-linked (pseudogene) |
| ITGB2-AS1 |  |  |  | ITGB2 antisense RNA 1 |
| IL7R |  |  |  | interleukin 7 receptor |
| PPIF |  |  |  | peptidylprolyl isomerase F |
| MAPK11 |  |  |  | mitogen-activated protein kinase 11 |
| ZNF204P |  |  |  | zinc finger protein 204, pseudogene |
| ZSCAN18 |  |  |  | zinc finger and SCAN domain containing 18 |
| NELL2 |  |  |  | neural EGFL like 2 |
| PLAG1 |  |  |  | PLAG1 zinc finger |
| APP |  |  |  | amyloid beta precursor protein |
| FCGBP |  |  |  | Fc fragment of IgG binding protein |
| ACSL6 |  |  |  | acyl-CoA synthetase long chain family member 6 |
| NAPRT |  |  |  | nicotinate phosphoribosyltransferase |
| ADA |  |  |  | adenosine deaminase |
| NPHP4 |  |  |  | nephrocystin 4 |
| CHAF1A |  |  |  | chromatin assembly factor 1 subunit A |
| MTRNR2L1 |  |  |  | MT-RNR2 like 1 |
| VSIG1 |  |  |  | V-set and immunoglobulin domain containing 1 |
| JUN |  |  |  | Jun proto-oncogene, AP-1 transcription factor subunit |
| SLC40A1 |  |  |  | solute carrier family 40 member 1 |
| HLA-DRA |  |  |  | major histocompatibility complex, class II, DR alpha |
| SH2D1A |  |  |  | SH2 domain containing 1A |
| LEF1 |  |  |  | lymphoid enhancer binding factor 1 |
| HLA-DQB1 |  |  |  | major histocompatibility complex, class II, DQ beta 1 |
| FOXP1 |  |  |  | forkhead box P1 |
| HLA-DRB1 |  |  |  | major histocompatibility complex, class II, DR beta 1 |
| CLIC1 |  |  |  | chloride intracellular channel 1 |
| SRSF8 |  |  |  | serine and arginine rich splicing factor 8 |
| RCAN3 |  |  |  | RCAN family member 3 |
| EIF3E |  |  |  | eukaryotic translation initiation factor 3 subunit E |
| PSMB3 |  |  |  | proteasome subunit beta 3 |
| CAMK4 |  |  |  | calcium/calmodulin dependent protein kinase IV |
| ST13 |  |  |  | ST13 Hsp70 interacting protein |
| SESN3 |  |  |  | sestrin 3 |
| ABI3 |  |  |  | ABI family member 3 |
| PTPN7 |  |  |  | protein tyrosine phosphatase non-receptor type 7 |
| BEX4 |  |  |  | brain expressed X-linked 4 |
| TIGIT |  |  |  | T cell immunoreceptor with Ig and ITIM domains |
| CTSC |  |  |  | cathepsin C |
| SAMD3 |  |  |  | sterile alpha motif domain containing 3 |
| ANXA2 |  |  |  | annexin A2 |
| SATB1 |  |  |  | SATB homeobox 1 |
| TRABD2A |  |  |  | TraB domain containing 2A |
| NAP1L1 |  |  |  | nucleosome assembly protein 1 like 1 |
| RNF213 |  |  |  | ring finger protein 213 |
| LAG3 |  |  |  | lymphocyte activating 3 |
| ALKBH7 |  |  |  | alkB homolog 7 |
| CD74 |  |  |  | CD74 molecule |
| CMC1 |  |  |  | C-X9-C motif containing 1 |
| GAPDH |  |  |  | glyceraldehyde-3-phosphate dehydrogenase |
| IFI16 |  |  |  | interferon gamma inducible protein 16 |
| GZMA |  |  |  | granzyme A |
| PABPC1 |  |  |  | poly(A) binding protein cytoplasmic 1 |
| EOMES |  |  |  | eomesodermin |

**Supplementary Table 8. List of overlapping DEGs in T-cells from patients with high vs low ApoB:ApoA1 vs human atherosclerotic plaque**

List of significantly differentially expressed genes (DEGs) from JSLE patients with high vs low ApoB:ApoA1 ratio, CD4^+^ T-cells (left) and CD8^+^ T-cells (right) and DEGs in T-cells from human atherosclerotic plaque. DEGs that overlap between the two groups are shown. Dark blue=significantly different. See Supplementary Figure 8e.


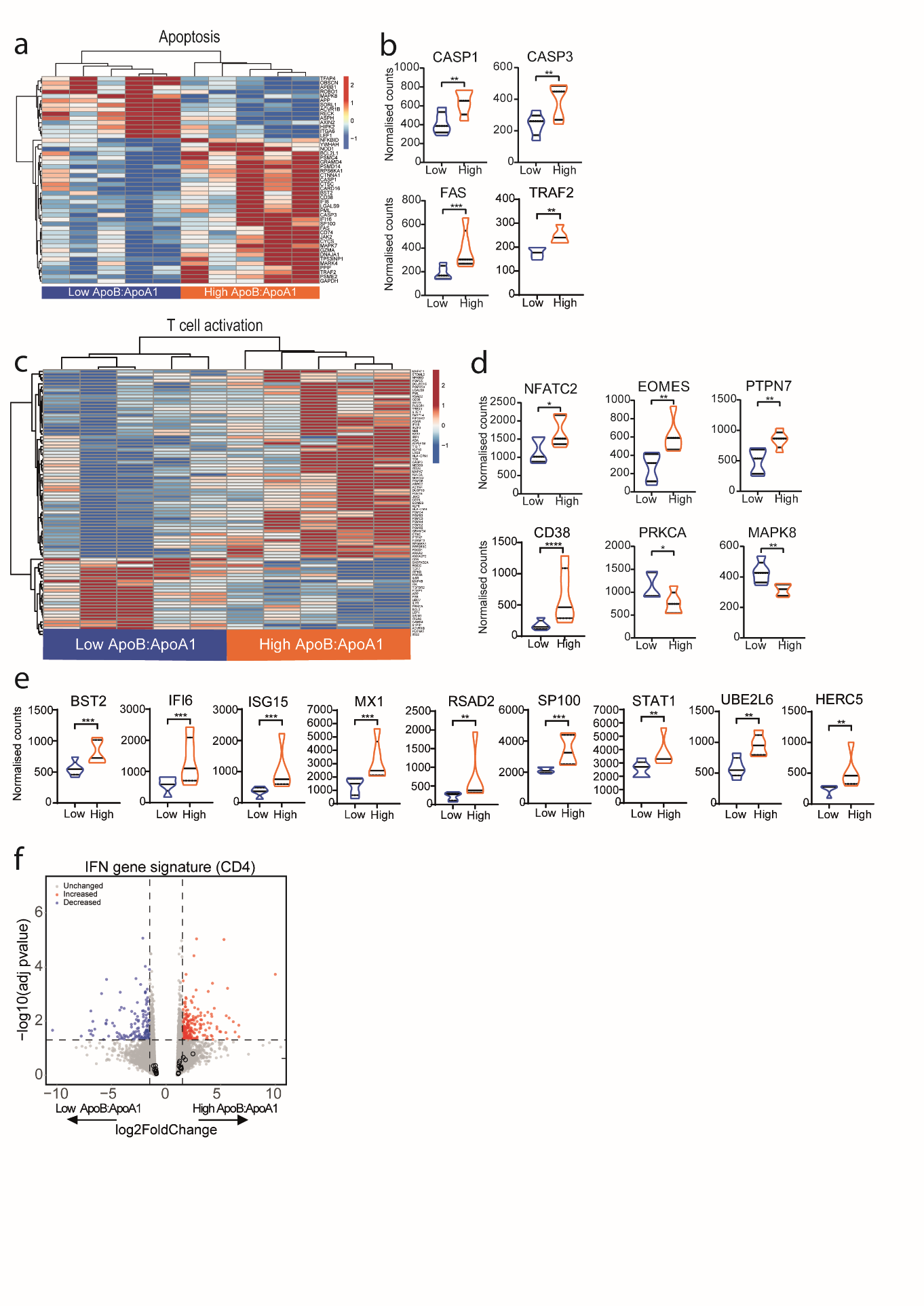
**Supplementary Figure 9.** **Genes associated with apoptosis, T-cell activation and interferon signalling altered between JSLE patients with high vs low ApoB:ApoA1 ratio.**

**Supplementary Figure 9.** **Genes associated with** **apoptosis, T-cell activation and interferon signalling significantly altered between JSLE patients with high vs low ApoB:ApoA1 ratio.** **(a)** Heatmap of normalised gene counts of DEGs in the ‘Apoptosis’ genetic pathway ontology term in (Figure 5a). Examples of genes significantly altered displayed as violin plots in **(b).**  **(c)** Heatmap displaying z scored normalised gene counts between high (n=5) and low (n=5) ApoB:ApoA1 ratio groups for CD8+ T-cell genes that contribute to the ‘T-cell activation’ genetic pathway ontology term (Supplementary Table 10). **(d)** Violin plots comparing normalised gene counts of genes associated with T-cell specific activation. **(e)** Violin plots comparing normalised CD8+ T-cell gene counts from JSLE patients with high vs low ApoB:ApoA1 (n=5/group) in the interferon signaling pathway Unpaired t test, *=P<0.05, **=P<0.01, ***=P<0.001, ****=P<0.0001. See Figure 5b. **(f)** Volcano plot (VolcaNoseR <https://huygens.science.uva.nl/VolcaNoseR>) showing genes associated with the Interferon (IFN) gene pathway ontology term identified in Figure 5a in CD4+ T-cells from JSLE patients with high vs low ApoB:ApoA1 ratio (n=5/group). Log2 Fold change and p-value plotted, coloured points represent significantly regulated genes. No genes overlap with the SLE-associated IFN signature described by El-Sherbiny YM and colleagues (5).

**Supplementary Table 9. DEGs in regulated pathways from CD8+ T-cells from JSLE patients with High vs Low ApoB:ApoA1 ratios and human and mouse atherosclerotic plaques.**

| **Summary** | **Pathways** | **LogP** | **Term** | **Genes** |
| --- | --- | --- | --- | --- |
| **Interferon** | **Interferon Signaling** | -20.14 | R-HSA-913531 | ADAR,B2M,BST2,CD44,EIF4A2,IFI6,HLA-A,HLA-DPA1,HLA-DPB1,HLA-DQA1,HLA-DQB1,HLA-DRA,HLA-DRB1,HLA-DRB5,IFI35,IFNGR1,IRF1,JAK1,JAK2,CIITA,MT2A,MX1,OAS3,PML,EIF2AK2,PTPN6,RPS27A,SP100,STAT1,STAT2,UBA52,UBC,TRIM25,IFITM1,OASL,EIF4G3,UBE2L6,ISG15,IRF9,IFITM2,USP18,SAMHD1,HERC5,RSAD2,EIF4E3 |
|  | **Type I interferon signaling pathway** | -14.71 | GO:  0060337 | ADAR,BST2,IFI6,IFI35,MX1,MYD88,OAS3,SP100,STAT1,STAT2,IFITM1,OASL,ISG15,IRF9,IFITM2,USP18,TREX1,ZBP1,RSAD2 |
| **T cell activation** | **T cell activation** | -27.48 | GO:  0042110 | ADA,ANXA1,RHOH,B2M,BCL2,ZFP36L1,ZFP36L2,CAMK4,CASP3,RUNX3,CD2,CD3D,CD3E,CD3G,CD6,CD7,CD8B,CD27,CD28,CD44,CD74,CCR7,CD55,FANCA,FCER1G,FYN,GPR18,HLA-DMB,HLA-DPA1,HLA-DPB1,HSPD1,IL6R,IL7R,IRF1,ITGAL,ITK,ITPKB,LAG3,LCK,LGALS3,LGALS9,SMAD7,NFATC2,PDCD1,PRKAR1A,PRKCQ,PTPN6,RPS3,RPS6,SATB1,CCL5,XCL1,SOD1,SP3,TCF7,PRDX2,TGFBR2,TP53,EOMES,TNFSF14,TNFRSF18,GRAP2,RIPOR2,BTN3A1,DUSP10,TREX1,PTPN22,LAT,FOXP1,STOML2,LEF1,PELI1,PREX1,BCL11B,TNFAIP8L2,DOCK8,NFKBID,RSAD2,TMIGD2,TIGIT,EIF2AK4,AP1G1,BST2,CD38,KLF6,FLT3LG,IGBP1,IGHG3,ITGA4,TYROBP,IRS2,TOX,IKZF3,TRDC,PPP2R3C,SLC39A10,SLAMF7,SAMSN1,DCLRE1C,ANXA2,ANXA2P2,APP,FOS,GLO1,GNAS,IFI16,JUN,JUNB,MYC,PDE1B,PRKCA,KLF10,SH3PXD2A,CTSC,FER,PLSCR1,CST7,JAK2,RPS6KA1,ACVR1B,H3-3A,H3-3B,HMGB2,HSPA1A,HSPA1B,PIM1,MAPK11,EIF2AK2,PSMA4,PSMA6,PSMB3,PSMC3,PSMC4,PSMC5,PSMD7,PSMD8,PSME2,STAT1,ISG15,PSMD14,N4BP2L2,GPR171,KLF13,SELL,SKAP1,FERMT3,ITGA6,MAPK7,RGCC,ACTN4,ATP5F1B,C1QBP,S1PR1,EPHA1,CXCR3,LMO7,NEDD9,PDE3B,PLXNA1,PML,RASA1,TBCD,CYTH1,ARPC2,ADAR,TSC22D3,PCBP2,RPS19,NMI,GRAMD4,SAMHD1,PARP14 |
|  | **Cytokine production** | -19.89 | GO:0001816 | ACP5,ADCY7,ABCD2,ANXA1,APP,B2M,BST2,C1QBP,CAMK4,CASP1,CD2,CD3E,CD247,CD6,CD28,CD74,CCR7,CD55,SERPINB1,FCER1G,GAPDH,GPR18,HLA-DPA1,HLA-DPB1,HMGB2,HSPA1A,HSPA1B,HSPB1,HSP90AB1,HSPD1,IFI16,IFNGR1,IL6R,IRF1,ITK,JAK2,LAG3,LGALS9,LTB,SMAD7,MYD88,NFATC2,PCBP2,PCSK5,PML,SRGN,PRKCQ,MAPK11,EIF2AK2,PTPN6,REL,RPS3,SARS1,XCL1,SOD1,STAT1,TIA1,TMSB4X,TRAF2,TXK,TYROBP,TRIM25,EOMES,NMI,UBE2L6,LITAF,ISG15,KLF2,NOD1,SPON2,BTN3A2,BTN3A1,TREX1,LSM14A,PTPN22,FOXP1,PDCD4,RGCC,NOP53,STOML2,LEF1,HERC5,DDX60,ASH1L,PELI1,HEG1,GPSM3,IFIH1,ZBP1,RSAD2,CARD16,TMIGD2,PYHIN1,TIGIT,MCOLN2 |
|  | **Th1 and Th2 cell differentiation** | -17.42 | hsa-04658 | RUNX3,CD3D,CD3E,CD3G,CD247,FOS,HLA-DMA,HLA-DMB,HLA-DPA1,HLA-DPB1,HLA-DQA1,HLA-DQB1,HLA-DRA,HLA-DRB1,HLA-DRB5,IFNGR1,JAK1,JAK2,JUN,LCK,NFATC2,NFKBIB,PRKCQ,MAPK1,MAPK8,MAPK11,STAT1,STAT4,LAT |
|  | **Regulation of innate immune response** | -15.15 | GO:  0045088 | ADAR,AP1G1,C1QBP,FCER1G,FYN,HMGB2,HSPA1A,HSPA1B,HSP90AB1,HSPD1,ICAM3,IFI16,IFNGR1,IRF1,JAK1,JAK2,LAG3,LGALS9,SH2D1A,MYD88,PLSCR1,PSMA4,PSMA6,PSMB3,PSMC3,PSMC4,PSMC5,PSMD7,PSMD8,PSME2,PTPN6,RPS19,RPS27A,CCL5,SKP1,STAT1,TXK,UBA52,UBC,UBE2D2,UBE2D3,NMI,PSMD14,NOD1,DUSP10,USP18,TREX1,TAB2,GRAMD4,SAMHD1,LSM14A,PTPN22,NOP53,PARP14,DDX60,PELI1,IFIH1,ZBP1,PARP9,RSAD2,PYHIN1,CD6,CD28,CTSC,CCR7,OPTN,PDCD4,GPSM3,NEAT1,CCL3L3,ACTN4,EZH2,FABP5,DNAJA1,PIM1,PML,PPP5C,RAN,SAFB,THRA,YWHAH,DHRS3,DDX17,UFL1,FOXP1,EGLN2 |
|  | **CD8 TCR downstream pathway** | -10.98 | M272 | B2M,CD3D,CD3E,CD3G,CD247,CD8B,FOS,HLA-A,JUN,JUNB,NFATC2,PRKCA,PRKCQ,MAPK1,MAPK8,PTPN7,STAT4,EOMES,TNFRSF18 |
| **Apoptosis** | **Apoptosis** | -13.21 | R-HSA-109581 | FAS,BCL2,BCL2L1,CASP3,HMGB2,LMNA,LMNB1,PLEC,PMAIP1,PRKCQ,MAPK1,MAPK8,PSMA4,PSMA6,PSMB3,PSMC3,PSMC4,PSMC5,PSMD7,PSMD8,PSME2,RPS27A,SATB1,TP53,TRAF2,UBA52,UBC,VIM,YWHAH,STK24,CFLAR,AKT3,PSMD14,CYCS |
|  | **Apoptotic signaling pathway** | -14.54 | GO:  0097190 | ACVR1B,ANXA6,FAS,BCL2,BCL2L1,BNIP3L,CASP1,CASP3,CD3E,CD27,CD28,CD38,CD44,CD74,CTSC,CTNNA1,CTSH,ENO1,ERN1,FYN,IFI6,HIP1,HINT1,HMGB2,DNAJA1,HSPA1A,HSPA1B,HSPB1,IFI16,IL6R,ITGA6,JAK2,JUN,LCK,LGALS3,LGALS9,LMNA,PMAIP1,PML,SRGN,PRKCA,MAPK7,MAPK8,RPL11,RPS3,RPS7,SKIL,SOD1,SP100,STK4,PRDX2,TFAP4,TP53,TPT1,TRAF2,TYROBP,YWHAH,DAP3,STK24,CFLAR,SLC9A3R1,PPIF,RACK1,ARL6IP5,ERP29,HIPK2,SHISA5,PLEKHF1,ITM2C,DAPL1,HIPK1,TLE5,BIN1,ANXA1,APBB1,BTG1,C1QBP,GAPDH,GZMA,HSPD1,ITGA4,MYC,GADD45B,PDCD1,ROBO1,RPS6,RPS27A,S100B,CCL5,SRPK2,TIA1,UBA52,UBC,ARHGEF1,NOD1,ANP32B,TXNIP,GRAMD4,PDCD4,RGCC,CYCS,PREX1,MARK4,OBSCN,NFKBID,TP53INP1,FOS,AXIN2,ASPH,IGBP1,RPS6KA1,CST7,TNFSF14,LEF1,CARD16,APP,PSME2,PSMD14,BST2,SERPINB1,PEBP1,SORL1,RECK,UBXN1,SMAD7,PSMC4,SUMO2,OGT,UBQLN2,NOP53,PYHIN1 |
|  | **Regulation of lymphocyte apoptotic process** | -10.62 | GO:  0070228 | ADA,CD3G,CD27,CD74,TSC22D3,IL7R,LGALS3,LGALS9,PDCD1,PRKCQ,CCL5,TP53,IRS2,FOXP1,SLC39A10,DOCK8,NFKBID |
|  | **Homeostasis of number of cells** | -14.82 | GO:  0048872 | ACVR1B,ADA,ADAR,ANXA1,B2M,BCL2,ZFP36L1,CASP3,CD74,CCR7,TSC22D3,EZH2,FCER1G,FLT3LG,HMGB2,HSPA1A,HSPA1B,IL7R,ITPKB,JAK2,LGALS9,PMAIP1,MAPK11,RPS6,RPS14,RPS17,RPS19,RPS24,SKIL,SOD1,SP3,STAT1,PRDX2,THRA,TNFSF14,ISG15,AKT3,KLF2,LAT,HIPK2,GCNT4,KLF13,PPP2R3C,ACTN1,ANXA2,ANXA2P2,APP,CAMK4,FOS,GLO1,GNAS,H3-3A,H3-3B,IFI16,JUN,JUNB,LGALS3,MYC,PDE1B,PML,PRKCA,PRKCQ,PTPN6,TGFBR2,KLF10,TYROBP,SH3PXD2A,WDR1,FOXP1,GPR171,LEF1 |
| **Other** | **Regulation of protein stability** | -11.28 | GO:  0031647 | ASPH,ATP1B3,BCL2,CAMLG,CASP3,CTSH,GAPDH,HIP1,HSPA1A,HSPA1B,HSPA8,HSP90AA1,HSP90AB1,HSPD1,LMNA,SMAD7,PFN1,PIM1,MAPK1,RPL5,RPL11,RPS7,STK4,TBL1X,TP53,CCT3,TYROBP,USP8,B4GALT5,RPL23,CCT7,CCT4,CCT2,CCT8,USP18,TREX1,HYPK,NOP53,TOMM7,ATF7IP,PYHIN1 |
|  | **Positive regulation of hydrolase activity** | -12.33 | GO:  0051345 | APP,FAS,ARHGAP5,ASPH,ATP1B3,CALM1,CALM3,CASP1,CCR7,CTSH,S1PR1,EPHA1,EZH2,FYN,HIP1,HMGB2,DNAJA1,HSPA1A,HSP90AB1,HSPD1,IFI16,ITGA6,ITK,JAK2,JUN,LCK,LGALS9,LLGL2,MYC,NEDD9,PFN1,PLSCR1,PMAIP1,PML,MAPK8,PSME2,RAB4A,RASA1,RASA2,RGS1,RGS10,ROBO1,RPS3,CCL4,CCL5,XCL1,XCL2,TBCD,TCEA1,TFAP4,TMSB4X,TRAF2,TXK,S1PR4,CFLAR,ARHGEF1,ACAP1,PSMD14,RASGRP2,DNAJA2,NOD1,RACK1,ANP32B,ARL6IP5,RAP1GAP2,GRAMD4,TBC1D1,ARHGAP45,DNAJB11,RAPGEF6,CYCS,SLC39A10,PREX1,DOCK8,ARAP2,TAGAP,AGRN,CCL3L3,RHOH,SOD1 |
|  | **Measles** | -14.36 | hsa-05162 | ADAR,FAS,CCND2,CD3D,CD3E,CD3G,CD28,FYN,HSPA1A,HSPA1B,HSPA8,IFNGR1,JAK1,JAK2,SH2D1A,MX1,MYD88,NFKBIB,OAS3,PRKCQ,EIF2AK2,STAT1,STAT2,TP53,EIF3H,AKT3,IRF9,RACK1,TAB2,IFIH1,EIF2AK4 |

**Supplementary Table 9. DEGs in regulated pathways from CD8+ T-cells from JSLE patients with High vs Low ApoB:ApoA1 ratios and human and mouse atherosclerotic plaques.** List of genes associated with significantly altered pathways that overlap between cardiovascular T-cell comparisons from Figure 5a.

**Supplementary Figure 10. Phenotypic summary of the stratified JSLE patient groups**


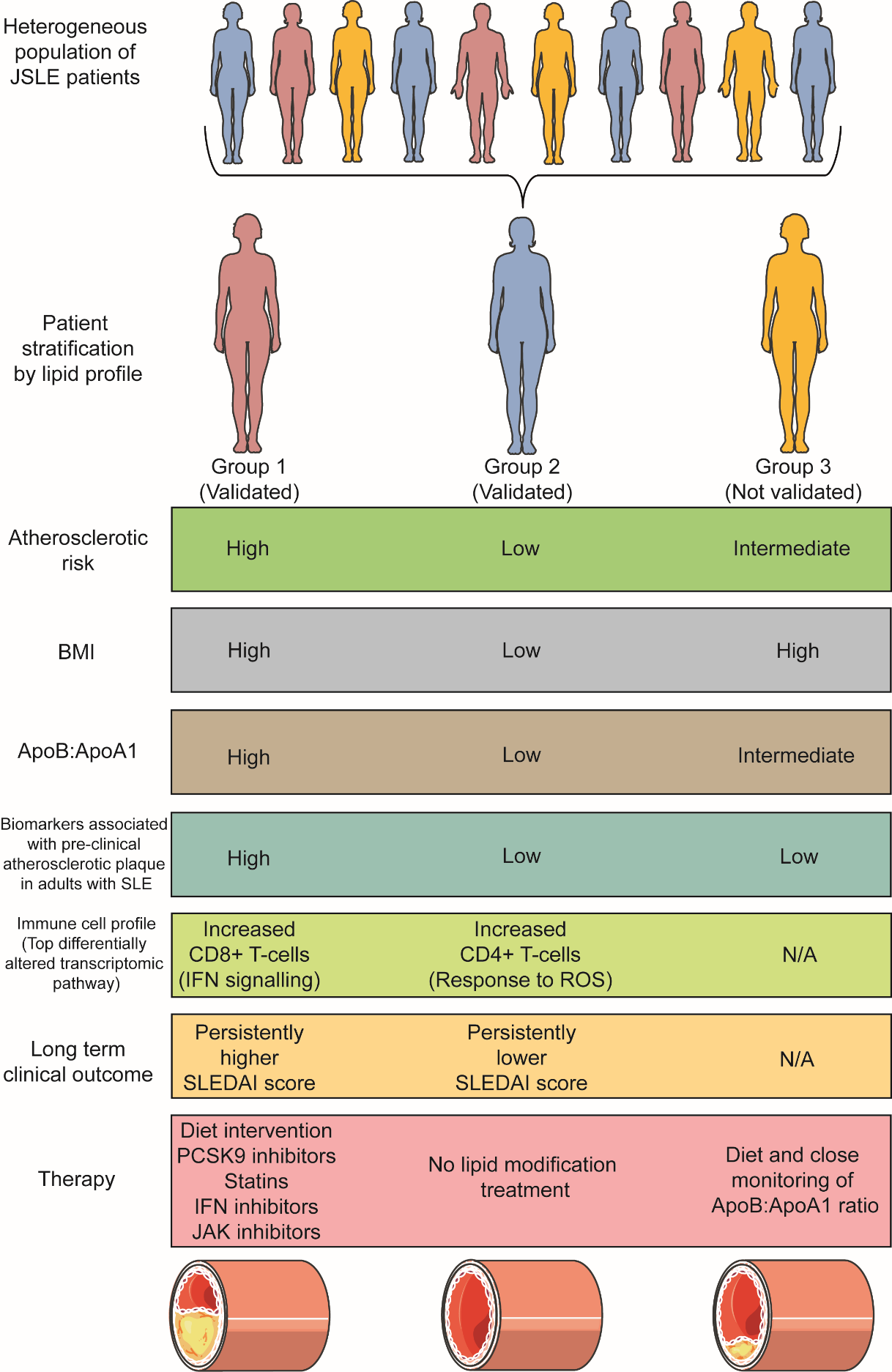


Supplementary Figure 10. Phenotypic summary of the stratified JSLE patient groups. Summary of the metabolic and immune cell phenotype as well as the proposed atherosclerotic risk and therapeutic options for lipid stratified JSLE patient groups. Therapeutic options include diet and/or therapies shown to be effective in large randomised trials in lowering cardiovascular disease risk. Abbreviations: Apo (Apolipoprotein), BMI (Body Mass Index), IFN (interferon), JAK (Janus kinase), PCSK9 (Pro-protein convertase subtilisin/kexin type 9), SLEDAI-2000 (Systemic Lupus Erythematosus Disease Activity Index). Images were provided from Servier Medical Art, licensed under a Creative Common Attribution 3.0 Generic License http://smart.servier.com.

**Supplementary Data Table**

**Data Table S1. Normalised logistic regression metabolomics data comparing Group-1/1A and Group-2/2A**

| **Metabolite** | **Group-1/1A (mean)** | **Group-2/2A (mean)** | **Normalised Odds ratio** | **Odds ratio SE** | **Odds ratio confidence intervals** | **P value** | **P value (corrected, Holm-Sidak)** | **Significantly affected by parameter** |
| --- | --- | --- | --- | --- | --- | --- | --- | --- |
| **Lipoproteins** |  |  |  |  |  |  |  |  |
| XXL-VLDL-P | 1.26E-11 | 9.45E-11 | 2.71 | 1.95 | 0.72 – 10.07 | 0.07088 | 0.99922 | BMI |
| XXL-VLDL-L | 2.61E-03 | 2.04E-02 | 2.73 | 1.97 | 0.72 – 10.25 | 0.07048 | 0.99922 | BMI |
| XXL-VLDL-PL | 2.47E-04 | 2.14E-03 | 2.14 | 1.86 | 0.63 – 7.18 | 0.14689 | 0.99999 | BMI |
| XXL-VLDL-C | 3.05E-04 | 4.24E-03 | 3.65E+01 | 6.00 | 1.08 – 1223.75 | 0.01993 | 0.90509 | BMI |
| XXL-VLDL-CE | 1.79E-04 | 2.71E-03 | 3.07E+01 | 4.65 | 1.5 – 625.54 | 0.00571 | 0.56901 | BMI |
| XXL-VLDL-FC | 1.25E-04 | 1.53E-03 | 2.33 | 1.93 | 0.64 – 8.47 | 0.11875 | 0.99991 | BMI |
| XXL-VLDL-TG | 2.06E-03 | 1.40E-02 | 2.63 | 1.92 | 0.73 – 9.48 | 0.07409 | 0.99933 | BMI |
| XL-VLDL-P | 4.66E-11 | 5.60E-10 | 2.63 | 2.01 | 0.66 – 10.33 | 0.09741 | 0.99982 | BMI |
| XL-VLDL-L | 4.40E-03 | 5.47E-02 | 2.66 | 2.02 | 0.66 – 10.58 | 0.09369 | 0.99982 | BMI |
| XL-VLDL-PL | 5.00E-04 | 8.39E-03 | 2.64 | 2.04 | 0.65 – 10.63 | 0.09579 | 0.99982 | BMI |
| XL-VLDL-C | 7.51E-04 | 1.21E-02 | 3.66 | 2.31 | 0.71 – 18.87 | 0.05077 | 0.99508 | BMI |
| XL-VLDL-CE | 5.19E-04 | 7.38E-03 | 4.67 | 2.51 | 0.77 – 28.27 | 0.03269 | 0.97365 | BMI |
| XL-VLDL-FC | 2.32E-04 | 4.67E-03 | 2.72 | 2.07 | 0.65 – 11.38 | 0.08720 | 0.99973 | BMI |
| XL-VLDL-TG | 3.15E-03 | 3.43E-02 | 2.50 | 1.98 | 0.65 – 9.54 | 0.11223 | 0.99990 | BMI |
| L-VLDL-P | 5.16E-10 | 4.27E-09 | 1.70E+01 | 3.95 | 1.14 – 251.54 | 0.01385 | 0.83045 |  |
| L-VLDL-L | 2.90E-02 | 2.46E-01 | 1.77E+01 | 4.02 | 1.15 – 271.13 | 0.01377 | 0.83045 |  |
| L-VLDL-PL | 4.70E-03 | 4.39E-02 | 2.08E+01 | 4.25 | 1.22 – 353.03 | 0.01208 | 0.79892 |  |
| L-VLDL-C | 5.79E-03 | 5.79E-02 | 2.43E+01 | 4.27 | 1.41 – 419.47 | 0.00761 | 0.64616 |  |
| L-VLDL-CE | 3.93E-03 | 3.47E-02 | 1.99E+01 | 3.46 | 1.74 – 227.52 | 0.00228 | 0.32489 |  |
| L-VLDL-FC | 1.87E-03 | 2.32E-02 | 3.27 | 2.17 | 0.71 – 14.93 | 0.05807 | 0.99748 | BMI |
| L-VLDL-TG | 1.85E-02 | 1.44E-01 | 1.45E+01 | 3.84 | 1.04 – 202.59 | 0.01831 | 0.89106 |  |
| M-VLDL-P | 5.80E-09 | 1.72E-08 | 5.89E+01 | 5.67 | 1.96 – 1766.17 | 0.00255 | 0.34376 |  |
| M-VLDL-L | 1.90E-01 | 5.73E-01 | 5.49E+01 | 5.49 | 1.95 – 1545.55 | 0.00242 | 0.33450 |  |
| M-VLDL-PL | 3.95E-02 | 1.16E-01 | 4.53E+01 | 4.98 | 1.94 – 1052.46 | 0.00199 | 0.29792 |  |
| M-VLDL-C | 4.63E-02 | 1.55E-01 | 2.07E+01 | 3.41 | 1.87 – 229.49 | 0.00133 | 0.22166 |  |
| M-VLDL-CE | 3.06E-02 | 9.12E-02 | 1.88E+01 | 3.23 | 1.89 – 187.25 | 0.00073 | 0.13894 |  |
| M-VLDL-FC | 1.56E-02 | 6.41E-02 | 4.57E+01 | 5.18 | 1.81 – 1149.87 | 0.00356 | 0.42887 |  |
| M-VLDL-TG | 1.04E-01 | 3.02E-01 | 6.77E+01 | 6.28 | 1.84 – 2480.23 | 0.00405 | 0.46261 |  |
| S-VLDL-P | 1.31E-08 | 2.96E-08 | 1.33E+01 | 2.79 | 1.78 – 99.07 | 0.00097 | 0.17356 |  |
| S-VLDL-L | 2.54E-01 | 5.74E-01 | 1.31E+01 | 2.75 | 1.8 – 94.94 | 0.00086 | 0.15895 |  |
| S-VLDL-PL | 6.55E-02 | 1.29E-01 | 9.66 | 2.54 | 1.54 – 60.23 | 0.00173 | 0.26870 |  |
| S-VLDL-C | 8.30E-02 | 1.98E-01 | 1.16E+01 | 2.45 | 2 – 67.41 | 0.00024 | 0.05143 |  |
| S-VLDL-CE | 4.97E-02 | 1.22E-01 | 1.48E+01 | 2.62 | 2.24 – 97.93 | 0.00011 | 0.02408 | BMI |
| S-VLDL-FC | 3.33E-02 | 7.61E-02 | 1.07E+01 | 2.60 | 1.64 – 69.91 | 0.00139 | 0.22844 |  |
| S-VLDL-TG | 1.06E-01 | 2.47E-01 | 1.35E+01 | 2.98 | 1.58 – 115.06 | 0.00234 | 0.32908 |  |
| XS-VLDL-P | 2.14E-08 | 3.46E-08 | 9.19 | 2.40 | 1.64 – 51.24 | 0.00103 | 0.18215 |  |
| XS-VLDL-L | 2.72E-01 | 4.33E-01 | 9.94 | 2.49 | 1.66 – 59.27 | 0.00095 | 0.17080 |  |
| XS-VLDL-PL | 8.57E-02 | 1.25E-01 | 1.00E+01 | 2.67 | 1.45 – 68.95 | 0.00224 | 0.32142 | BMI |
| XS-VLDL-C | 1.29E-01 | 2.00E-01 | 1.58E+01 | 3.06 | 1.76 – 140.91 | 0.00065 | 0.12580 | BMI |
| XS-VLDL-CE | 8.38E-02 | 1.34E-01 | 1.70E+01 | 3.17 | 1.77 – 163.71 | 0.00060 | 0.11839 |  |
| XS-VLDL-FC | 4.50E-02 | 6.66E-02 | 1.07E+01 | 2.76 | 1.46 – 78.8 | 0.00210 | 0.31100 | BMI |
| XS-VLDL-TG | 5.78E-02 | 1.08E-01 | 8.94 | 2.62 | 1.35 – 59.12 | 0.00439 | 0.48541 |  |
| IDL-P | 6.34E-08 | 8.73E-08 | 1.10E+01 | 2.98 | 1.29 – 93.39 | 0.00233 | 0.32908 | BMI |
| IDL-L | 6.42E-01 | 8.75E-01 | 1.13E+01 | 3.13 | 1.2 – 105.55 | 0.00316 | 0.40134 | BMI |
| IDL-PL | 1.87E-01 | 2.41E-01 | 1.12E+01 | 3.26 | 1.1 – 113.08 | 0.00554 | 0.56080 | BMI |
| IDL-C | 3.88E-01 | 5.27E-01 | 8.53 | 2.93 | 1.03 – 69.96 | 0.00755 | 0.64616 | BMI |
| IDL-CE | 2.66E-01 | 3.76E-01 | 1.16E+01 | 3.29 | 1.11 – 119.57 | 0.00392 | 0.45842 | BMI |
| IDL-FC | 1.22E-01 | 1.52E-01 | 3.73 | 2.13 | 0.84 – 16.45 | 0.04250 | 0.98907 | BMI |
| IDL-TG | 6.75E-02 | 1.06E-01 | 4.52 | 2.09 | 1.07 – 19.08 | 0.01261 | 0.81041 |  |
| L-LDL-P | 1.03E-07 | 1.43E-07 | 1.29E+01 | 3.27 | 1.27 – 131.57 | 0.00264 | 0.34996 | BMI |
| L-LDL-L | 7.38E-01 | 1.01 | 1.36E+01 | 3.42 | 1.21 – 151.14 | 0.00323 | 0.40514 | BMI |
| L-LDL-PL | 2.07E-01 | 2.61E-01 | 7.63 | 2.68 | 1.1 – 52.81 | 0.00686 | 0.62105 | BMI |
| L-LDL-C | 4.74E-01 | 6.69E-01 | 2.47E+01 | 4.90 | 1.09 – 555.36 | 0.00399 | 0.46182 | BMI |
| L-LDL-CE | 3.17E-01 | 4.74E-01 | 1.11E+02 | 9.99 | 1.22 – 10139.15 | 0.00216 | 0.31703 | BMI |
| L-LDL-FC | 1.57E-01 | 1.95E-01 | 4.66 | 2.28 | 0.92 – 23.46 | 0.02497 | 0.94400 | BMI |
| L-LDL-TG | 5.73E-02 | 8.59E-02 | 4.05 | 2.02 | 1.02 – 16.03 | 0.01836 | 0.89106 | BMI |
| M-LDL-P | 8.06E-08 | 1.15E-07 | 1.66E+01 | 3.59 | 1.36 – 203 | 0.00239 | 0.33315 | BMI |
| M-LDL-L | 4.15E-01 | 5.86E-01 | 2.00E+01 | 4.02 | 1.3 – 305.69 | 0.00259 | 0.34638 | BMI |
| M-LDL-PL | 1.26E-01 | 1.61E-01 | 7.56 | 2.54 | 1.21 – 46.99 | 0.00446 | 0.48849 | BMI |
| M-LDL-C | 2.61E-01 | 3.85E-01 | 3.89E+01 | 5.58 | 1.33 – 1127.89 | 0.00245 | 0.33621 | BMI, Mycophenolate, Azathioprine |
| M-LDL-CE | 1.66E-01 | 2.71E-01 | 4.62E+01 | 5.70 | 1.52 – 1396.57 | 0.00164 | 0.25896 | BMI, Mycophenolate, Azathioprine |
| M-LDL-FC | 9.47E-02 | 1.14E-01 | 5.80 | 2.44 | 1 – 33.32 | 0.01680 | 0.87765 | BMI |
| M-LDL-TG | 2.81E-02 | 4.06E-02 | 3.31 | 1.93 | 0.91 – 12 | 0.03596 | 0.98013 | BMI |
| S-LDL-P | 9.76E-08 | 1.28E-07 | 9.13 | 3.03 | 1.03 – 80.35 | 0.01401 | 0.83092 | BMI |
| S-LDL-L | 2.76E-01 | 3.59E-01 | 8.98 | 3.06 | 1 – 80.47 | 0.01623 | 0.87070 | BMI |
| S-LDL-PL | 9.79E-02 | 1.09E-01 | 2.90 | 2.04 | 0.71 – 11.75 | 0.11207 | 0.99990 | BMI |
| S-LDL-C | 1.63E-01 | 2.26E-01 | 1.77E+01 | 4.38 | 0.97 – 320.05 | 0.01158 | 0.78770 | BMI |
| S-LDL-CE | 1.06E-01 | 1.61E-01 | 3.23E+01 | 5.56 | 1.11 – 932.81 | 0.00602 | 0.58513 | BMI |
| S-LDL-FC | 5.69E-02 | 6.52E-02 | 3.08 | 2.02 | 0.77 – 12.2 | 0.08374 | 0.99966 | BMI |
| S-LDL-TG | 1.56E-02 | 2.37E-02 | 3.40 | 1.94 | 0.93 – 12.39 | 0.02928 | 0.96305 | BMI |
| XL-HDL-P | 5.01E-07 | 2.01E-07 | 2.48E-01 | 1.87 | 0.07 – 0.84 | 0.00332 | 0.41092 |  |
| XL-HDL-L | 5.03E-01 | 2.00E-01 | 2.60E-01 | 1.84 | 0.07 – 0.86 | 0.00406 | 0.46261 |  |
| XL-HDL-PL | 2.77E-01 | 1.01E-01 | 2.16E-01 | 1.99 | 0.05 – 0.82 | 0.00217 | 0.31703 |  |
| XL-HDL-C | 2.15E-01 | 8.77E-02 | 1.88E-01 | 2.07 | 0.04 – 0.78 | 0.00128 | 0.21532 |  |
| XL-HDL-CE | 1.57E-01 | 6.84E-02 | 1.82E-01 | 2.10 | 0.04 – 0.77 | 0.00122 | 0.20736 |  |
| XL-HDL-FC | 5.75E-02 | 1.93E-02 | 4.71E-02 | 3.49 | 0 – 0.54 | 0.00029 | 0.06020 |  |
| XL-HDL-TG | 1.08E-02 | 1.05E-02 | 1.06 | 1.57 | 0.43 – 2.55 | 0.90473 | 1.00000 | BMI |
| L-HDL-P | 1.44E-06 | 6.69E-07 | 8.44E-02 | 2.40 | 0.01 – 0.46 | 0.00008 | 0.01842 |  |
| L-HDL-L | 9.16E-01 | 4.14E-01 | 8.29E-02 | 2.41 | 0.01 – 0.46 | 0.00008 | 0.01782 |  |
| L-HDL-PL | 4.20E-01 | 2.02E-01 | 9.24E-02 | 2.31 | 0.01 – 0.47 | 0.00008 | 0.01842 |  |
| L-HDL-C | 4.65E-01 | 1.85E-01 | 7.42E-02 | 2.52 | 0.01 – 0.45 | 0.00007 | 0.01677 |  |
| L-HDL-CE | 3.62E-01 | 1.48E-01 | 7.54E-02 | 2.51 | 0.01 – 0.45 | 0.00007 | 0.01675 |  |
| L-HDL-FC | 1.03E-01 | 3.67E-02 | 7.12E-02 | 2.58 | 0.01 – 0.45 | 0.00008 | 0.01767 |  |
| L-HDL-TG | 3.04E-02 | 2.71E-02 | 5.04E-01 | 1.80 | 0.15 – 1.59 | 0.23028 | 1.00000 |  |
| M-HDL-P | 1.85E-06 | 1.38E-06 | 3.28E-02 | 4.06 | 0 – 0.51 | 0.00108 | 0.18781 | BMI |
| M-HDL-L | 7.89E-01 | 5.80E-01 | 3.73E-02 | 3.78 | 0 – 0.5 | 0.00088 | 0.16180 |  |
| M-HDL-PL | 3.59E-01 | 2.69E-01 | 4.70E-02 | 3.50 | 0 – 0.54 | 0.00114 | 0.19531 |  |
| M-HDL-C | 4.03E-01 | 2.74E-01 | 6.84E-02 | 2.85 | 0 – 0.53 | 0.00042 | 0.08464 |  |
| M-HDL-CE | 3.33E-01 | 2.29E-01 | 6.23E-02 | 2.94 | 0 – 0.51 | 0.00039 | 0.07991 |  |
| M-HDL-FC | 7.01E-02 | 4.58E-02 | 1.03E-01 | 2.47 | 0.01 – 0.6 | 0.00059 | 0.11681 |  |
| M-HDL-TG | 2.65E-02 | 3.65E-02 | 2.30 | 1.77 | 0.75 – 7.03 | 0.09560 | 0.99982 | BMI |
| S-HDL-P | 4.36E-06 | 4.37E-06 | 9.39E-01 | 1.72 | 0.32 – 2.71 | 0.90069 | 1.00000 | BMI |
| S-HDL-L | 9.70E-01 | 9.66E-01 | 8.70E-01 | 1.74 | 0.29 – 2.57 | 0.78680 | 1.00000 | BMI |
| S-HDL-PL | 5.31E-01 | 5.02E-01 | 3.25E-01 | 2.08 | 0.07 – 1.36 | 0.10947 | 0.99990 | BMI |
| S-HDL-C | 4.04E-01 | 4.07E-01 | 1.15 | 1.79 | 0.36 – 3.61 | 0.78984 | 1.00000 | BMI |
| S-HDL-CE | 3.04E-01 | 3.13E-01 | 1.39 | 1.80 | 0.43 – 4.36 | 0.54870 | 1.00000 | BMI |
| S-HDL-FC | 1.00E-01 | 9.47E-02 | 3.45E-01 | 1.99 | 0.08 – 1.32 | 0.10755 | 0.99990 | BMI |
| S-HDL-TG | 3.47E-02 | 5.66E-02 | 1.86E+01 | 3.16 | 1.95 – 177.14 | 0.00091 | 0.16605 |  |
| **Lipoprotein diameters** |  |  |  |  |  |  |  |  |
| VLDL-D | 3.52E+01 | 3.67E+01 | 4.16 | 1.89 | 1.19 – 14.45 | 0.00621 | 0.59241 |  |
| LDL-D | 2.36E+01 | 2.36E+01 | 2.20 | 1.84 | 0.66 – 7.27 | 0.11180 | 0.99990 | BMI |
| HDL-D | 1.01E+01 | 9.68 | 3.64E-02 | 3.71 | 0 – 0.47 | 0.00017 | 0.03603 |  |
| **General cholesterol** |  |  |  |  |  |  |  |  |
| Serum-C | 3.04 | 3.39 | 1.86 | 1.73 | 0.63 – 5.43 | 0.26900 | 1.00000 | BMI |
| VLDL-C | 2.68E-01 | 6.30E-01 | 1.63E+01 | 3.01 | 1.88 – 141.68 | 0.00068 | 0.13160 |  |
| Remnant-C | 6.55E-01 | 1.16 | 1.18E+01 | 2.66 | 1.74 – 80.32 | 0.00055 | 0.11013 | BMI |
| LDL-C | 8.99E-01 | 1.28 | 3.17E+01 | 5.53 | 1.1 – 906.62 | 0.00400 | 0.46182 | BMI |
| HDL-C | 1.49 | 9.57E-01 | 6.26E-02 | 2.85 | 0 – 0.48 | 0.00015 | 0.03189 |  |
| HDL2-C | 1.02 | 5.06E-01 | 7.73E-02 | 2.56 | 0.01 – 0.48 | 0.00013 | 0.02798 |  |
| HDL3-C | 4.70E-01 | 4.51E-01 | 1.05E-01 | 3.73 | 0 – 1.38 | 0.03442 | 0.97725 | BMI, dsDNA |
| Esterified-C | 2.14 | 2.35 | 1.66 | 1.72 | 0.57 – 4.78 | 0.38143 | 1.00000 | BMI |
| Free-C | 9.04E-01 | 1.04 | 2.49 | 1.83 | 0.76 – 8.11 | 0.10924 | 0.99990 | BMI |
| **Glycerides and phospholipids** |  |  |  |  |  |  |  |  |
| Serum-TG | 5.76E-01 | 1.25 | 1.50E+01 | 3.43 | 1.34 – 168.24 | 0.00749 | 0.64551 |  |
| VLDL-TG | 3.05E-01 | 8.53E-01 | 4.72E+01 | 5.58 | 1.62 – 1373.27 | 0.00601 | 0.58513 |  |
| LDL-TG | 1.01E-01 | 1.52E-01 | 3.69 | 1.97 | 0.97 – 13.95 | 0.02368 | 0.93646 | BMI |
| HDL-TG | 1.02E-01 | 1.42E-01 | 4.95 | 2.33 | 0.93 – 26.07 | 0.02550 | 0.94601 | BMI |
| TotPG | 1.37 | 1.37 | 4.43E-01 | 1.86 | 0.13 – 1.49 | 0.15584 | 0.99999 | BMI |
| PC | 1.39 | 1.42 | 5.81E-01 | 1.81 | 0.18 – 1.85 | 0.34671 | 1.00000 | BMI |
| SM | 3.33E-01 | 3.41E-01 | 1.00 | 1.78 | 0.32 – 3.09 | 0.99538 | 1.00000 | BMI |
| TG/PG | 6.40E-01 | 3.30E-01 | 1.75E+01 | 3.15 | 1.83 – 165.73 | 0.00113 | 0.19502 | BMI |
| Tot-cholines | 1.71 | 1.73 | 5.91E-01 | 1.78 | 0.19 – 1.82 | 0.33333 | 1.00000 | BMI |
| **Apolipoproteins** |  |  |  |  |  |  |  |  |
| ApoA1 | 1.44 | 1.21 | 2.45E-01 | 1.80 | 0.07 – 0.77 | 0.00142 | 0.23131 |  |
| ApoB | 5.28E-01 | 7.72E-01 | 9.73 | 2.49 | 1.63 – 58 | 0.00085 | 0.15801 | BMI |
| ApoB/ApoA1 | 3.69E-01 | 6.43E-01 | 1.64E+01 | 2.72 | 2.31 – 116.4 | 0.00012 | 0.02680 |  |
| **Fatty Acid ratios** |  |  |  |  |  |  |  |  |
| TotFA | 7.32 | 9.02 | 2.28 | 1.78 | 0.73 – 7.04 | 0.11326 | 0.99990 | BMI |
| UnSat | 1.24 | 1.19 | 3.63E-01 | 1.98 | 0.09 – 1.39 | 0.09651 | 0.99982 | BMI |
| DHA/FA | 9.32E-02 | 9.66E-02 | 2.88E-01 | 2.08 | 0.06 – 1.2 | 0.04192 | 0.98885 | BMI |
| LA/FA | 2.25 | 2.46 | 2.79E-01 | 2.06 | 0.06 – 1.15 | 0.04260 | 0.98907 |  |
| FAw3/FA | 2.79E-01 | 3.12E-01 | 4.09E-01 | 2.00 | 0.1 – 1.58 | 0.14397 | 0.99998 | BMI |
| FAw6/FA | 2.69 | 2.93 | 2.44E-01 | 2.03 | 0.06 – 0.97 | 0.01733 | 0.88351 |  |
| PUFA/FA | 2.97 | 3.24 | 2.49E-01 | 2.02 | 0.06 – 0.98 | 0.01794 | 0.89011 |  |
| MUFA/FA | 1.76 | 2.58 | 4.23 | 1.89 | 1.21 – 14.7 | 0.00634 | 0.59704 |  |
| SFA/FA | 2.59 | 3.20 | 6.36E-01 | 1.71 | 0.22 – 1.81 | 0.38637 | 1.00000 | BMI |
| **Glycolysis metabolites** |  |  |  |  |  |  |  |  |
| Glc | 3.51 | 3.58 | 2.49E-01 | 2.37 | 0.04 – 1.35 | 0.09244 | 0.99982 | BMI |
| Lac | 1.86 | 1.80 | 6.58E-01 | 1.80 | 0.2 – 2.08 | 0.46969 | 1.00000 |  |
| Pyr | 7.11E-02 | 6.65E-02 | 4.48E-01 | 1.67 | 0.16 – 1.22 | 0.09264 | 0.99982 | BMI |
| Cit | 1.13E-01 | 1.11E-01 | 6.19E-01 | 1.63 | 0.23 – 1.6 | 0.27648 | 1.00000 | BMI |
| Glol | 8.73E-02 | 9.05E-02 | 2.03 | 1.93 | 0.55 – 7.36 | 0.22295 | 1.00000 |  |
| **Amino Acids** |  |  |  |  |  |  |  |  |
| Ala | 3.94E-01 | 3.74E-01 | 6.20E-01 | 1.60 | 0.24 – 1.56 | 0.26739 | 1.00000 |  |
| Gln | 4.42E-01 | 4.22E-01 | 1.10 | 1.70 | 0.39 – 3.12 | 0.84082 | 1.00000 | BMI |
| Gly | 2.81E-01 | 2.81E-01 | 1.85 | 1.73 | 0.62 – 5.41 | 0.18245 | 1.00000 | BMI |
| His | 6.74E-02 | 6.42E-02 | 5.83E-01 | 1.89 | 0.16 – 2.04 | 0.34268 | 1.00000 | BMI |
| Ile | 4.23E-02 | 5.13E-02 | 8.74E-01 | 1.69 | 0.31 – 2.43 | 0.83648 | 1.00000 | BMI |
| Leu | 6.12E-02 | 6.74E-02 | 8.41E-01 | 1.61 | 0.32 – 2.14 | 0.70365 | 1.00000 | BMI |
| Val | 1.40E-01 | 1.51E-01 | 7.76E-01 | 1.63 | 0.29 – 2.01 | 0.55944 | 1.00000 | BMI |
| Phe | 6.88E-02 | 7.86E-02 | 1.46 | 1.68 | 0.53 – 4.02 | 0.51941 | 1.00000 |  |
| Tyr | 5.46E-02 | 5.19E-02 | 5.17E-01 | 1.65 | 0.19 – 1.38 | 0.12329 | 0.99993 | BMI |
| **Other** |  |  |  |  |  |  |  |  |
| Acetate | 5.40E-02 | 5.84E-02 | 1.57 | 1.61 | 0.62 – 3.98 | 0.30566 | 1.00000 | BMI |
| Acetoacetate | 3.99E-02 | 4.28E-02 | 5.91E-01 | 1.90 | 0.16 – 2.08 | 0.49245 | 1.00000 | BMI |
| bOHBut | 1.19E-01 | 1.31E-01 | 1.08 | 1.58 | 0.44 – 2.66 | 0.87162 | 1.00000 | BMI |
| Crea | 4.94E-02 | 5.41E-02 | 8.72E-01 | 1.70 | 0.3 – 2.46 | 0.81790 | 1.00000 | BMI |
| Alb | 9.06E-02 | 8.97E-02 | 1.26 | 1.84 | 0.37 – 4.15 | 0.68563 | 1.00000 | BMI |
| Gp | 1.18 | 1.48 | 1.09E+01 | 2.86 | 1.39 – 85.68 | 0.00322 | 0.40514 |  |

**Data Table S1. Normalised logistic regression metabolomics data comparing Group-1/1A and Group-2/2A.** Table displaying the mean value of metabolites in the validation and discovery cohort combined groups (Group-1/1A and Group-2/2A) as well as data from logistic regression analysis of each metabolite between the groups including odds ratios, odds ratio SE, p values, p values corrected for multiple testing (Holm-Sidak) and clinical/demographic parameters that significantly influence the metabolite during normalisation. Significant values between groups are shown in red.

**Supplementary Data Table S2:** **Logistic regression immune phenotype data comparing Group-1/1A and Group-2/2A**

| Immune cell | OR | 95% CI | p_value |
| --- | --- | --- | --- |
| CD4+ | 0.905491 | 0.83259- 0.984776 | 0.02044 |
| CD4+ CM | 1.05251 | 0.968811- 1.14344 | 0.22607 |
| CD4+ EM | 0.996855 | 0.844253-1.177041 | 0.97036 |
| CD4+ EMRA | 1.053108 | 0.358166-3.096434 | 0.92508 |
| CD4+ Naive | 0.968009 | 0.90609-1.034159 | 0.33501 |
| CD8+ | 1.12064 | 1.023077-1.227506 | 0.01425 |
| CD8+ CM | 1.148304 | 1.018115-1.295141 | 0.0243 |
| CD8+ EM | 1.190111 | 0.932937-1.518178 | 0.16117 |
| CD8+ EMRA | 0.993116 | 0.921366-1.070453 | 0.85672 |
| CD8+ Naive | 0.959938 | 0.903803-1.01956 | 0.18354 |
| iNKT | 2.818113 | 6.32E-06-1256559 | 0.87594 |
| Treg | 1.110786 | 0.712739-1.731132 | 0.64256 |
| Tresp | 1.03567 | 0.94933-1.129862 | 0.43002 |
| CD19+ | 1.205196 | 0.967556-1.501203 | 0.09578 |
| Bm1 | 0.915286 | 0.770225-1.087667 | 0.31467 |
| Bm2 | 1.015345 | 0.959333-1.074628 | 0.59889 |
| Bm2 (Transitional) | 1.162344 | 0.975837-1.384498 | 0.09182 |
| Bm3-Bm4 | 1.441282 | 0.577525-3.596888 | 0.43339 |
| Early Bm5 | 0.958385 | 0.869821-1.055965 | 0.39022 |
| Late Bm5 | 0.907273 | 0.771019-1.067605 | 0.24117 |
| CD19+ Naive | 1.032681 | 0.979447-1.088808 | 0.23368 |
| CD19+ Switched memory | 0.951408 | 0.86349-1.048278 | 0.31397 |
| CD19+ Unswitched memory | 0.871135 | 0.714742-1.061749 | 0.17178 |
| CD14+ | 0.984261 | 0.86202-1.123838 | 0.81463 |
| Classical | 0.944451 | 0.779041-1.144984 | 0.56072 |
| Intermediate | 1.126428 | 0.728321-1.742144 | 0.59258 |
| non-classical | 0.888246 | 0.650715-1.212483 | 0.45541 |
| PDC's | 0.004765 | 1.14E-05-1.993321 | 0.08256 |

**Supplementary Data Table 2:** **Logistic regression immune phenotype data comparing Group-1/1A and Group-2/2A.** Peripheral blood mononuclear cells (PBMC’s) from high (Group1/1A, n=29) and low (Group 2/2A, n=25) ApoB:ApoA1 ratio JSLE patients were stained ex-vivo to evaluate expression of 28 immune cell subsets by flow cytometry. Logistic regression Odds ratios (OR), 95% confidence intervals (CIs) and P value of 28 immunological parameters were computed with univaraite logistic regression analysis adjusted for BMI, sex, age, ethnicity, disease duration, treatment, SLEDAI-2000, CRP, dsDNA, C3. Refer to Figure 4a.

References

1. Hochberg MC. Updating the American college of rheumatology revised criteria for the classification of systemic lupus erythematosus. Arthritis & Rheumatism. 1997;40(9):1725-.

2. Petri M, Orbai A-M, Alarcón GS, Gordon C, Merrill JT, Fortin PR, et al. Derivation and validation of the Systemic Lupus International Collaborating Clinics classification criteria for systemic lupus erythematosus. Arthritis & Rheumatism. 2012;64(8):2677-86.

3. Yee C-S, Farewell VT, Isenberg DA, Griffiths B, Teh L-S, Bruce IN, et al. The use of Systemic Lupus Erythematosus Disease Activity Index-2000 to define active disease and minimal clinically meaningful change based on data from a large cohort of systemic lupus erythematosus patients. Rheumatology. 2010;50(5):982-8.

4. Franklyn K, Lau CS, Navarra SV, Louthrenoo W, Lateef A, Hamijoyo L, et al. Definition and initial validation of a Lupus Low Disease Activity State (LLDAS). Annals of the Rheumatic Diseases. 2016;75(9):1615-21.

5. El-Sherbiny YM, Psarras A, Yusof MYM, Hensor EMA, Tooze R, Doody G, et al. A novel two-score system for interferon status segregates autoimmune diseases and correlates with clinical features. Scientific Reports. 2018;8:11.
